# Adherence to breast cancer guidelines is associated with better survival outcomes: a systematic review and meta-analysis of observational studies in EU countries

**DOI:** 10.1186/s12913-020-05753-x

**Published:** 2020-10-07

**Authors:** Ignacio Ricci-Cabello, Adrián Vásquez-Mejía, Carlos Canelo-Aybar, Ena Niño de Guzman, Javier Pérez-Bracchiglione, Montserrat Rabassa, David Rigau, Ivan Solà, Yang Song, Luciana Neamtiu, Elena Parmelli, Zuleika Saz-Parkinson, Pablo Alonso-Coello

**Affiliations:** 1 Balearic Islands Health Research Institute (IdISBa), Palma, Spain; 2 Primary Care Research Unit of Mallorca, Balearic Islands Health Service, Palma, Spain; 3 CIBER de Epidemiología y Salud Pública (CIBERESP), Madrid, Spain; 4 Facultad de Medicina Humana, Universidad Nacional Mayor de San Marcos, Lima, Peru; 5 Iberoamerican Cochrane Centre - Department of Clinical Epidemiology and Public Health, Biomedical Research Institute Sant Pau (IIB Sant Pau), Sant Antonio María Claret 167, 08025 Barcelona, Spain; 6 Interdisciplinary Centre for Health Studies (CIESAL), Universidad de Valparaíso, Valparaíso, Chile; 7 European Commission, Joint Research Centre (JRC), Ispra, Italy

**Keywords:** Breast cancer, Clinical guidelines, Adherence, Survival, Systematic review

## Abstract

**Background:**

Breast cancer (BC) clinical guidelines offer evidence-based recommendations to improve quality of healthcare for patients with or at risk of BC. Suboptimal adherence to recommendations has the potential to negatively affect population health. However, no study has systematically reviewed the impact of BC guideline adherence -as prognosis factor- on BC healthcare processes and health outcomes. The objectives are to analyse the impact of guideline adherence on health outcomes and on healthcare costs.

**Methods:**

We searched systematic reviews and primary studies in MEDLINE and Embase, conducted in European Union (EU) countries (inception to May 2019). Eligibility assessment, data extraction, and risk of bias assessment were conducted by one author and crosschecked by a second. We used random-effects meta-analyses to examine the impact of guideline adherence on overall survival and disease-free survival, and assessed certainty of evidence using GRADE.

**Results:**

We included 21 primary studies. Most were published during the last decade (90%), followed a retrospective cohort design (86%), focused on treatment guideline adherence (95%), and were at low (80%) or moderate (20%) risk of bias. Nineteen studies (95%) examined the impact of guideline adherence on health outcomes, while two (10%) on healthcare cost. Adherence to guidelines was associated with increased overall survival (HR = 0.67, 95%CI 0.59–0.76) and disease-free survival (HR = 0.35, 95%CI 0.15–0.82), representing 138 more survivors (96 more to 178 more) and 336 patients free of recurrence (73 more to 491 more) for every 1000 women receiving adherent CG treatment compared to those receiving non-adherent treatment at 5 years follow-up (moderate certainty). Adherence to treatment guidelines was associated with higher costs, but adherence to follow-up guidelines was associated with lower costs (low certainty).

**Conclusions:**

Our review of EU studies suggests that there is moderate certainty that adherence to BC guidelines is associated with an improved survival. BC guidelines should be rigorously implemented in the clinical setting.

**Trial registration:**

PROSPERO (CRD42018092884).

## Background

Breast cancer is the world’s most common cancer in women with an estimated 2.08 million new cancer cases diagnosed in 2018, accounting for 24.2% of all cancers [1]. The illness is diagnosed more frequently in developed countries. According to the European Cancer Information System, more than 400,000 incident female breast cancer cases and 98,000 deaths were estimated in the European Union (EU28) for 2018 [2] (Additional file 1). Preventing, diagnosing and treating breast cancer is, therefore, an important priority for health policymakers. Treatment procedures have changed dramatically over recent years. As new and more precise diagnostic strategies have shown, treatment for early and metastatic breast cancer has improved [3–7]. Similarly, advances in breast cancer screening and treatment have reduced breast cancer mortality across the age spectrum in the past decade [8–10].

Clinical guidelines (CGs) are statements that include recommendations intended to optimise patient care [11]. Evidence-based high-quality CGs are becoming increasingly available as a result of recent advances in health services research [12]. However, the effectiveness of the CGs heavily depends not only on their quality but also on how they are implemented, and embedded in clinicians´ routine clinical practice [13].

Since CGs recommendations should be based on best available evidence, adherence to CGs is expected to result in better patient outcomes. However, most CGs rely on evidence from clinical trials, which are usually performed with relatively small samples at sites with experienced investigators and highly selected participants, and therefore are at risk of overestimating benefits and underestimating harms [14]. Moreover, they are generally conducted in high-income countries, and, as a consequence, tend to be very resource-intensive [15]. Therefore, the external validity and clinical utility of their recommendations must be confirmed in real clinical practice. Moreover, non-adherence can be due to valid reasons (mainly related to contraindications and patient preferences), and therefore many CGs deviations are intentional and do not necessarily impact negatively on the quality of care [16].

Some studies have shown that CGs adherence can have a positive impact on patient outcomes in the field of breast cancer [17–20]. On the other hand, some authors have suggested a paradox by which guideline deviations would contribute to increased survival of patients with breast cancer (proposing that observed improvements in health outcomes during the last two decades may be more due to higher resource availability rather than adherence to guidelines) [21, 22]. To the best of our knowledge, no previous systematic review has examined the impact of breast cancer CGs adherence on health outcomes.

Adherence to breast cancer CGs might behave as a prognostic factor for health outcomes. A prognosis factor is any measure that, among people with a given health condition, is associated with a subsequent clinical outcome [23]. Even though most researched prognostic factors are biomarkers, or patient’s characteristics, these may also be measured outside the individual, at an ecological level, such as health care access, quality of care, and breast cancer guidelines adherence in which the exposure of individuals is inferred [23]. The objective of this systematic review is two-fold: i) to identify if adherence (compared to non-adherence) to CGs impacts on patient-related outcomes and ii) to identify if adherence (compared to non-adherence) to CGs impacts on healthcare costs.

## Methods

We conducted a systematic review following the standard Cochrane Collaboration methods [24] and adhering to the PRISMA statement reporting guidelines [25]. We registered the research protocol in PROSPERO (CRD42018092884).

### Data sources and searches

We designed search strategies (May 2019) to search Embase (accessed through Ovid) and MEDLINE (PubMed). In a first step we focused the search to identify systematic reviews as a source of primary studies, and then we searched primary studies. Additional file 2 shows the search algorithms.

### Eligibility of studies

We used the PICO (*P*opulation, *I*ntervention, *C*omparison, *O*utcome) framework to guide our eligibility criteria (Table 1). We included observational studies (before-after, cohort, case-control, and cross-sectional studies) examining the impact of CGs adherence on: 1) breast cancer patient-related outcomes (overall survival, disease-free survival, quality of life, incidence-based mortality, harm), and 2) healthcare costs. We included studies conducted only in the 27 countries members of the European Union at the time the review was conducted (as this systematic review was conducted within the European Commission Initiative on Breast Cancer). Multicounty studies conducted in EU and non-EU countries were excluded. The list of the 27 countries included is available in Additional file 3. We only included studies published in English. First, for calibration purposes two reviewers (IRC and AVM) independently screened 20% of the search results based on title and abstract and selected the references to be assessed based on full text. Then, the remaining 80% of the references were screened by a senior systematic reviewer (IRC). All references selected for full-text assessment were double-checked by a second author (AVM). The level of agreement between both reviewers was 85%. Disagreements were solved by discussion or with the help of a third author (ENDG).

**Table 1:** Structured clinical question

Population	Intervention	Comparison	Outcomes^a^
Healthcare professionals involved in breast cancer care (all processes)	Adherence to breast cancer CGs in the process of care	Non-adherence to breast cancer CGs in the process of care.	1) Impact on patient-related outcomes
			•Overall survival
			•Disease-free survival
			•Quality of life
			•Incidence based-mortality
			•Harm
			2) Impact on health care costs

^a^*List of prioritised outcomes produced as a result of agreement between JRC experts and CCIB members*

### Data extraction and risk of bias assessment

We extracted and described in tables, the main characteristics of included studies (e.g. country, publication year, guideline scope, study design, aim, and year of study, number of patients, patient’s characteristics, and adherence definition), and of the outcomes of interest. Data extraction was conducted in pairs, one author (IR) conducted the first extraction, subsequently, a second author (JPB) cross-checked the extracted data. If necessary, the corresponding authors of the studies included were contacted to retrieve further information.

For risk of bias assessment, we used specific tools depending on the study design. For longitudinal studies (including both prospective and retrospective follow-up designs) we used the Newcastle - Ottawa Quality Assessment Scale [26], which contains nine items classified in three main domains: selection, comparability, and outcome. The studies are graded according to a score from 0 (highest risk of bias) to 9 (lowest risk of bias). The following cut-off points were applied: < 5, high risk of bias; 5–7, moderate risk of bias; > 7, low risk of bias. For non-controlled before-after studies, we used the Quality Assessment Tool for Before-After (Pre-Post) Studies With No Control Group [27]; this tool includes 12 criteria, each of them rated as presence or absence of risk of bias. One author (IRC) carried out the risk of bias assessment, and a second author (AVM) cross-checked this assessment. Disagreements were solved by discussion or with the help of a third author.

### Data synthesis and analysis

Survival measures (overall survival and disease-free survival) and impact on health cost were compared between adherent and non-adherent groups of patients. For survival outcomes, only studies reporting HRs using Cox adjusted models with at least 5 years of follow up were pooled, using the Der Simonian and Laird random effects model [28]. Statistical heterogeneity was assessed using Cochran Q and I^2^ measure. An I^2^ value above 50 and 75% was predefined as moderate and high heterogeneity, respectively [29]. If selected studies had a similar source of data, (i.e. the BRENDA cohort [30]) to avoid the risk of double counting populations or events, we selected the cohort values with the longest follow-up and with more complete data reporting. Results were expressed as HR and the related 95% CI, a HR < 1 denote advantage for adherence and HR > 1 denotes advantage for non-adherence to CGs. *The anticipated absolute effects estimates were measured as risk differences. We collected the mortality and recurrence rates for a follow-up period of 60 months in both the intervention (*i.e. *adherent to CGs*) *and control (*i.e. *non-adherent to CGs) groups. The estimation of risk differences was performed indirectly from the pooled hazard ratios and the estimation of the baseline risk. For the latter, data was obtained from Kaplan Meier survival curves from the control groups* [31]*. We used Review Manager v.5.3* [32] *for the estimation of pooled hazard ratios, and we used GRADEpro* [33] *for the estimation of the absolute difference of events. We narratively synthesised findings for impact on healthcare costs, and survival outcomes for the remaining studies not included in the meta-analysis.*

To assess the certainty of evidence we applied the Grading of Recommendations Assessment, Development and Evaluation (GRADE) approach [12]. We downgraded the certainty of the evidence based on the assessment of: study limitations, inconsistency of results, indirectness of evidence, imprecision, and reporting bias. GRADE principles apply also to prognostic questions [34]. In contrast to treatment effects questions, in prognosis questions, cohorts with a prospective design, initially provide high certainty as they enable optimal measurement of predictors and outcomes [35]. Quality of evidence is classified in four categories: high (which indicates we are very confident that the true prognosis lies close to that of the estimate); moderate (we are moderately confident that the true prognosis is likely to be close to the estimate, but there is a possibility that it is substantially different); low (our confidence in the estimate is limited: the true prognosis may be substantially different), and; very low quality (any estimate of effect is very uncertain) [34]. We report the main findings both narratively and as tabulated summaries.

## Results

### Search results

**Fig. 1:**
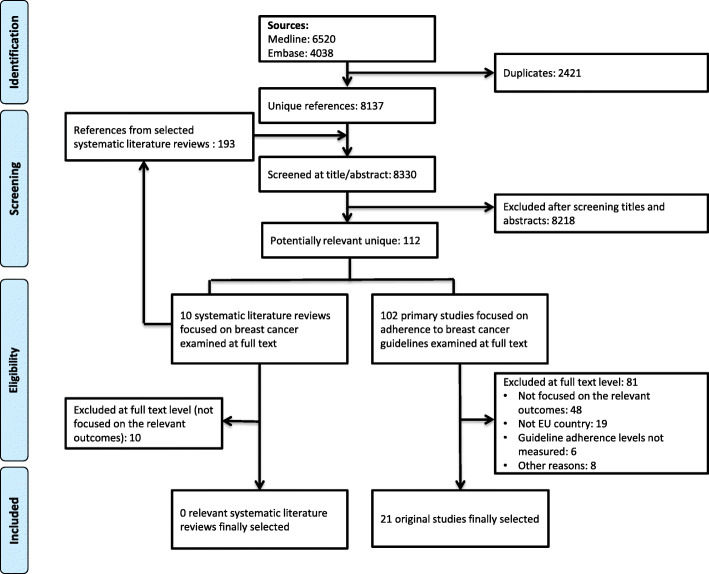
PRISMA flowchart describing selection of included systematic reviews and original studies

The eligibility process of the original studies is summarised in a PRISMA flowchart (Fig. 1). We retrieved a total of 8137 unique citations from database searches, which were reviewed along with another 193 references identified from ten identified systematic literature reviews. We selected 112 references for review at the full-text level. Of these, we excluded 91 publications (reasons for exclusion available in Additional file 3), and finally included 21 studies [17–22, 30, 36–49].

### Characteristics of the included studies

Most of included studies were conducted in Germany (15/21; 71%) and were published during the last decade (19/21, 90%). Most were retrospective cohort studies (18/21, 86%); of these, 13 were single cohorts (BRENDA study [30]) but included different periods and/or addressed different clinical questions; three were non-controlled before-after studies. Two studies examined the impact of adherence to CGs on healthcare costs, whereas 20 studies examined the impact on health outcomes (most frequently survival related measures). (Tables 2a and 2b, Additional file 4). No studies were identified examining the rest of the outcomes considered in this review (quality of life, incidence-based mortality, and harm).

**Table 2a:** Characteristics of the included studies (*n* = 21)

Study characteristics	n (%)	References
Country		
France	2 (10%)	[38, 39]
Germany	15 (71%)	[18–22, 30, 37, 41–43, 45–49]
Italy	2 (10%)	[17, 40]
Netherlands	2 (10%)	[36, 44]
Publication year		
2015–2019	8 (35%)	[17–22, 45, 49]
2009–2014	11 (52%)	[30, 40–44] [37, 39, 46–48]
≤ 2008	2 (10%)	[36, 38]
Study design		
Non-controlled before-after study	3 (14%)	[21, 38, 40]
Retrospective cohort study	18 (86%)	[17–20, 22, 30, 36, 37, 39, 41–49]
Guideline scope		
Diagnosis	2 (10%)	[17, 40]
Follow-up	1 (5%)	[38]
Treatment	20 (95%)	[17–22, 30, 36, 37, 39–49]
Outcomes‡		
Overall survival	18 (86%)	[12–17, 24–27, 29, 31–37, 49]
Disease free survival	16 (76%)	[13–15, 17, 24, 26, 28, 29, 31–37, 49]
Costs associated to CGs adherence	2 (10%)	[38, 39]
Quality of life	0 (0%)	–
Incidence-based mortality	0 (0%)	–
Harm	0 (0%)	–
Risk of bias		
Low	17 (81%)	[17, 20–22, 36, 37, 39–44, 46–49]
Moderate	4 (20%)	[18, 19, 38, 45]
High	0 (0%)	–

‡ Percentages exceed 100% because the categories are not mutually exclusive (i.e. some studies involved more than one type of guideline and more than one type of outcome)

**Table 2b:** Characteristics of the clinical practice guidelines examined by the included studies

Author/Year/Reference	Scope of the guideline(s)	Type of health care recommendation adherence was studied	Guidelines studied
Andreano 2017 [17]	National (Italian) guideline and European guidelines*	Diagnosis and treatment (generic)	NICE guideline a; ESMO guideline b
de Roos 2005 [36]	National (Dutch)	Treatment (treatment of patients with DCIS)	Otter 2003c
Ebner, Hancke 2015 [18]	National (German)	Treatment (generic)	S3 guideline d
Ebner 2015 [19]	National (German)	Treatment (generic)	S3 guideline d
Hancke 2010 [37]	National (German) and international guidelines*	Treatment (generic)	2005 St Gallen consensus e; S3 guideline d
Jacke 2015 [21]	National (German)	Treatment (generic)	S3 guideline d
Mille 2000 [38]	Regional (French)	Follow-up (post therapeutic follow-up)	Centre Régional Léon Bérard guidelines f
Poncet 2009 [39]	National and regional (French) guidelines	Breast cancer treatment (specific for trastuzumab treatment)	French post licensing guidelines (2001), regional clinical guidelines published by the regional oncology care network called “Convergence” in the French Rhone-Alpes area (no additional information provided).
Sacerdote 2013 [40]	Regional (Italy) guidelines	Treatment (generic)	Piedmont Clinical Practice Guideline g
Schwentner 2012 [43]	National (German)	Treatment (adjuvant)	S3 guideline d
Schwentner 2012 [42]	National (German)	Treatment (generic)	S3 guideline d
Schwentner 2013 [41]	National (German)	Treatment (generic)	S3 guideline d
Van de Water 2012 [44]	National (Dutch)	Treatment (guidelines for breast and axillary surgery, radiotherapy, chemotherapy and endocrine therapy)	Dutch guideline h
Van Ewijk 2015 [45]	National (German)	Treatment (adjuvant treatment)	S3 guideline d
Varga 2010 [46]	National (German)	Treatment (treatment of early-onset breast cancer)	S3 guideline d
Wollschlager 2017 [20]	National (German)	Treatment (guidelines for adjuvant treatment)	S3 guideline d
Wockel, Kurzeder et al. 2010 [48]	National (German) and international guidelines*	Treatment (generic)	2005 St Gallen consensus e; S3 guideline d
Wockel, Varga et al. 2010 [47]	National (German) and international guidelines*	Treatment (generic)	2005 St Gallen consensus e; S3 guideline d
Wolters 2015 [22]	National (German)	Treatment (adjuvant treatment)	S3 guideline d
Wockel 2014 [30]	National (German)	Treatment (generic)	S3 guideline d
Wimmer 2019 [49]	National (German)	Treatment (radiotherapy)	S3 guideline d

* adherence to recommendations from two different guidelines examined in this study

a, Early and locally advanced breast cancer diagnosis and treatment: full guideline. National Collaborating Centre for Cancer, Cardiff https://www.nice.org.uk/guidance/cg80/resources/early-and-locallyadvanced- breast-cancer-diagnosis-and- reatment-975,682,170,565

b, Senkus E, Kyriakides S, Ohno S et al. (2015) Primary breast cancer: ESMO Clinical Practice Guidelines for diagnosis, treatmentand follow-up. Ann Oncol 26:v8–v30. doi:10.1093/annonc/ mdv298

c, Otter R (ed) (2003) Richtlijnen voor diagnostiek en behandeling van premaligne en maligne aandoeningen in de IKN-regio 2003 pp. 338–339. Groningen: IKN; ISBN 90–74,114–25-3

d, Kreienberg K, Kopp I, Lorenz et al. Interdisciplinary S3 Guidelines for the diagnosis and treatment of breast cancer in women. German Cancer Society. 2004

e, Goldhirsch A, Glick JH, Gelber RD et al. Meeting highlights: international expert consensus on the primary therapy of early breast cancer 2005. Ann Oncol 2005; 16: 1569–1583. 9

f, Centre Régional Léon Bérard, Réseau Oncora: The’saurus ONCORA en Cancérologie. Paris, France, Arnette Blackwell, 1997, p 374

g, Regione Piemonte Assessorato Sanità, Commissione Oncologica Regionale, Centro di Riferimento per l’Epidemiologia e la Prevenzione Oncologica in Piemonte: Tumore della mammella - linee guida clinico organizzative per la

Regione Piemonte. 2002

h, Oncoline. *Dutch National Breast Cancer Guidelines*. http://www.oncoline.nl/mammacarcinoom

Almost all studies (*n* = 20) examined the impact of adherence to treatment guidelines, two also addressed adherence to diagnostic guidelines and one addressed adherence to guidelines for follow-up (Table 3).

**Table 3:** Summary of findings

Outcomes	№ of participants (studies) Follow-up	Certainty of the evidence (GRADE)	Relative effect (95% CI)	Anticipated absolute effects	
				Risk with Non-adherence to CG*	Risk difference with adherence to CG
Overall survival assessed with: adjusted hazard ratio follow up: median 60 months^a^	15,974 (4 non-randomised studies) [17, 40, 49, 50]	⨁⨁⨁◯ MODERATE ^a,b^	**HR 0.67** (0.59 to 0.76) [death]	431 per 1.000 ^c^	**138 more per 1.000** (96 more to 178)
Disease free survival assessed with: adjusted hazard ratio follow up: median 60 months^a^	9224 (3 non-randomised studies) [36, 49, 50]	⨁⨁◯◯ LOW ^a,b,d^	**HR 0.35** (0.15 to 0.82) [recurrence]	370 per 1.000 ^e^	**336 more per 1.000** (73 moreto 491 more)
Treatment costs^f^	(1 observational study) [39]	⨁⨁◯◯ LOW ^f,^	Higher costs for treatment concordant to CG.		
Follow-up costs^f^	(1 observational study) [38]	⨁⨁◯◯ LOW ^f,^	Non-CGs adherent follow-up was 2.2 to 3.6 times more expensive than the compliant one [5, 7].		

*The risk in the intervention group (and its 95% confidence interval) is based on the assumed risk in the comparison group and the relative effect of the intervention (and its 95% CI)

*CI* Confidence interval, *HR* Hazard Ratio

a) To assess this outcome we considered adherence to clinical guidelines as a prognosis factor for survival related outcomes. According to GRADE guidance for the assessment of prognosis clinical questions, observational studies are the main source of evidence, and have to potential to provide a high level of certainty. However, the optimal study design for prognosis questions are high quality prospective cohort studies. Although all the included studies used regression analyses to adjust for most important confounding clinical variables, we rated down our confidence in effects due concerns of serious risk of bias related with the study design (all selected studies were retrospective cohorts based on medical records or hospital database registries)

b) Despite the existence of important differences across studies both in terms of the context of application, and of the conceptualisation of adherence applied, we did not rate down for indirectness, as results were highly consistent across different contexts and definitions

c) The mortality rate in the group not receiving CG adherent treatment was 43.14% (1477/3424)

d) We rated down one level our confidence in effects due to the high level of unexplained heterogeneity (I2: 96%). However, it should be noted that in the context of guideline development, it may not be appropriate to rate down for heterogeneity, since the desirable effects are highly consistent across studies

e) Th recurrence rate in the group not receiving CG adherent treatment was 37% (578 / 1553)

f) To assess the impact of adherence on costs we used an intervention (rather than a prognosis) approach, since cost is not a clinical outcome and the objective of this assessment was to examine a potential causal relationship. For this reason, the certainty of evidence was rated as “low”, as only observational studies were available

GRADE Working Group grades of evidence

High certainty: We are very confident that the true effect lies close to that of the estimate of the effect

Moderate certainty: We are moderately confident in the effect estimate: The true effect is likely to be close to the estimate of the effect, but there is a possibility that it is substantially different

Low certainty: Our confidence in the effect estimate is limited: The true effect may be substantially different from the estimate of the effect

Very low certainty: We have very little confidence in the effect estimate: The true effect is likely to be substantially different from the estimate of effect

### Risk of bias assessment

Out of the 21 studies included, 17 presented low risk of bias [17, 20–22, 36, 37, 39–44, 46–49] and four presented moderate risk of bias [18, 19, 38, 45]. The most common reasons for risk of bias were related to the source of the non-exposed cohort, outcome assessment, and adequacy of follow-up (Tables 2a and 2b, Additional file 4).

### Impact on patient outcomes

#### Overall survival

**Fig. 2:**
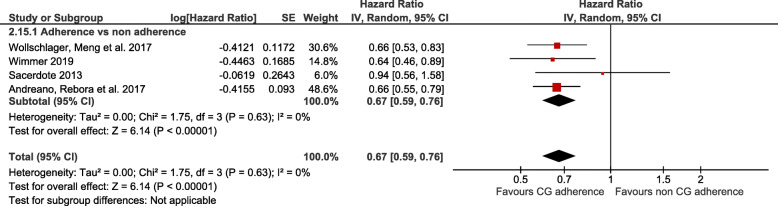
Random effect meta-analysis of the association between adherence to breast cancer clinical guidelines with overall survival rate

The random effects meta-analysis of four studies [17, 40, 49, 50] with 15,974 patients followed up for a median of 5 years (Fig. 2, Table 3), showed that adherence to breast cancer CGs was associated with better overall survival rates (HR = 0.67 (95% CI 0.59 to 0.76), I^2^: 0%). This means that for every 1000 patients, 129 more patients (from 91 more to 165 more) would potentially survive in the group of patients managed in compliance with breast cancer CGs, compared to those who were not (non-adherent group). The certainty of evidence for adherence to CGs as a prognosis factor for overall survival was moderate due to risk of bias (studies relied only on the accuracy of medical records).

Additionally, another 18 studies not meeting the criteria for performing a meta-analysis as specified in the methods section, assessed this outcome. Their characteristics and main results are available in Additional files 4 and 5. A direct association between adherence to breast cancer CGs and better overall survival was more frequently observed regardless of the study period [22], the use of different cut-offs or number of deviations to CGs recommendations used to determine adherence [17, 42, 48] and patient’s age [17, 18, 37, 41, 44, 45]. Although non-adherence was associated with lower survival rate both in triple negative breast cancer (TNBC) and non-TNBC patients, this association was stronger in TNBC than in non-TBNC patients [19, 43]. Seven studies explored adherence by treatment modality [18, 19, 30, 37, 43, 47, 48], revealing that there was better overall survival in patients receiving CG-compliant treatments for breast-conserving therapy, chemotherapy, endocrine therapy, and radiotherapy. One before after study did not find significant differences in survival rates comparing adherent versus non-adherent groups [21].

#### Disease-free survival

**Fig. 3:**
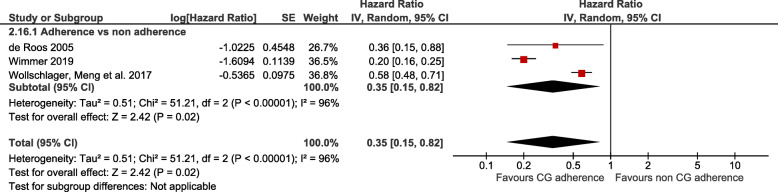
Random effect meta-analysis of the association between adherence to breast cancer clinical guidelines with disease-free survival

The random effects meta-analysis of three studies followed up a median of 5 years with 9224 patients [20, 36, 49] (Fig. 3, Table 3) showed better disease-free survival rates in patients receiving CG recommended treatment compared to those not receiving CG recommended treatment (HR = 0.35 (95% CI from 0.15 to 0.82); I^2^ = 96%). This means that for every 1000 women there were 336 more women free of recurrence (73 more to 491 more) in patients managed in compliance with breast CGs compared to those who were not. The certainty of evidence for adherence to breast cancer CGs as a prognosis factor for disease-free survival was moderate. As in overall survival, we considered rating down our confidence by one level for risk of bias for the same reason (Table 3).

Additionally, this outcome was reported in another 15 studies not meeting the criteria for performing a meta-analysis as specified in the methods section. An association between adherence to breast cancer CGs and better disease-free survival was observed consistently regardless of the study period [22, 47], use of different cut-offs to determine adherence [43, 47, 48], patient’s age [18, 41] and subtype of tumour [42, 43]. Subgroup analyses showed that adherence to recommendations in CGs was directly associated with better (*p* < 0.05) disease free-survival for most modalities of therapy [18, 19, 30, 47, 48]. Women over 70 years of age less often (*p* < 0.05) received recommended breast-conserving therapy (70–79 years: 74–83%; > 79 years: 54%) than women aged ≤69 years (93%) [37] (Additional file 5). The included studies did not report relevant data for the rest of the patient-important outcomes of interest (quality of life, incidence based-mortality, and harm).

### Impact on health care cost

Poncet et al. (2009) observed that treatment costs with trastuzumab per patient and per year were higher for patients receiving CGs-concordant treatment than for patients receiving CGs discordant treatment (EUR 54975 vs. EUR 44186, respectively). The main reason for this difference was the treatment cost since the type of molecules for combination with trastuzumab recommended in the CGs were more expensive than other alternatives not stated in the CGs [39]. Mille et al. (2000) observed that, in patients with localised breast cancer, the expenditure associated to CGs concordant healthcare (consultations and examinations) was consistently lower (from 2.2 to 3.6 times lower) compared to CGs discordant healthcare. Unjustified examinations mainly explained this difference [38]. The certainty of the evidence for the impact on health care costs was low both for adherence to treatment guidelines and for adherence to follow-up guidelines because in both cases only observational studies were available (Table 3, Additional file 4).

## Discussion

### Main findings

In this systematic review and meta-analysis, we observed that there is moderate certainty that adherence to breast cancer CGs by healthcare providers is associated with a substantial increase in overall survival and disease-free survival. We observed that for every 1000 women managed according to CGs compared to those that were not (non-adherent), there were 138 more survivors and 336 more free of recurrence patients over 5 years of follow-up.

### Our results in the context of previous research

Previous systematic reviews have shown that adherence to breast cancer CGs [51] and other types of cancer CGs [52, 53] remains suboptimal. Sustainable use of CGs is also notably suboptimal, with studies showing up to a 50% decrease in adherence after 1 year of implementation [54]. Suboptimal adherence to CGs could increase healthcare costs if healthcare resources are overused (e.g. overtreatment, overuse of diagnostic or screening techniques), but also if they are underused (i.e. increased costs to cover the additional healthcare needs that people may face with worsening conditions due to provision of inadequate care). In our review, we identified no solid evidence about the economic impact of non-adherence to breast cancer CGs. This finding supports the results from a previous systematic review, which noted an absence of robust economic evaluations of the impact of CGs implementation [55].

As far as we know, no previous systematic review has examined the impact of breast cancer CGs adherence as a prognosis factor of health outcomes. Our findings, however, resonate with those from a previous systematic review of adherence to gynaecologic cancer surgery CGs, which suggested that adoption of CGs was an effective tool for disease control; noting that CGs adherence should be considered as a process measure of quality cancer care [56]. A number of non-condition specific systematic reviews have evaluated the effectiveness of CGs [57–61]. These reviews (which differed in terms of population, interventions, and outcomes considered) show a trend that supports that CGs improve patient outcomes. However, they consistently noted that the amount and quality of evidence upon which to develop their conclusions were very poor and that more methodologically robust studies are needed to build a stronger evidence base around the impact of CGs adherence.

### Strengths and limitations

Our review has several strengths. We followed a robust methodology to produce quantitative estimates of the impact of CGs adherence, conducting meta-analyses, evaluating for the first time, the impact of adherence to breast cancer CGs on patient-important health outcomes (i.e., outcomes that patients value directly, instead of surrogate, outcomes that clinicians may consider important) [62]. We conducted detailed data extraction from selected studies and used specific tools for risk of bias assessment. We followed best practice for evidence assessment, using summary of finding tables and GRADE to assess the certainty of effects [63].

Our study has some limitations. First, we searched two bibliographic databases and only one author conducted the initial screening based on titles and abstracts (i.e., only 20% of the references were cross-checked by a second reviewer). Although data extraction and risk of bias assessment process was conducted in pairs using a cross-checking strategy rather than conducting them independently. by; these aspects could have limited our ability to identify additional studies. However, we included studies from four countries in the EU, with consistent findings. It is therefore unlikely that additional studies would have substantially changed the results. Second, we restricted the selection of studies to those published in English and in EU countries (due to the scope of the study), and most (71%) of the studies identified were conducted in a single country (Germany). This may limit the generalisability of our findings, which may not represent other contexts with different CGs development methods or implementation strategies. And third, since we framed our question as a prognosis question, our conclusions can only suggest an association rather than imply causality. Moreover, the absolute effect size of the risk of death or recurrence expressed should be interpreted with caution. The number of events will vary greatly depending on the time chosen; in this work we selected the estimates at 5 years of follow up. The estimation of absolute effect estimates was calculated indirectly using the pooled hazard ratios adjusted for prognostic factors and the number of events in the control groups. We are aware that selecting different baseline risks according to different settings or types of population would vary substantially the number of events.

### Implications for practice and research

The evidence identified in our review supports, with moderate certainty, that adherence to breast cancer CGs is associated with greater survival and disease-free survival. In light of our findings, the use of CGs should be more widely implemented. However, the way in which guideline adherence and implementation should be effectively enhanced remains uncertain: although several strategies have been proposed, and educational interventions are promising [64, 65], the evidence base about the efficacy of the proposed interventions is still limited [66]. Addressing this knowledge gap should be a priority for future research [67–69].

Our study provides valuable information, especially for health policymakers, to support the development of strategies to implement CGs and/or reinforce CGs-adherence in breast cancer care. Greater use of explicit theory to understand barriers, design interventions, and explore mediating pathways and moderators is also much needed to advance in this area [70, 71]. In addition, the certainty of evidence in relation to the impact of adherence to CGs on health care cost was low, and the available information is based on studies published more than a decade ago, when the cost of Trastuzumab was considerably more expensive than nowadays. Robust economic studies evaluating the impact of adherence to CGs on health care costs are needed to progress in this area.

## Conclusion

Our systematic review of studies conducted in the EU shows that there is moderate certainty that adherence to breast cancer guidelines is associated with improved overall and disease-free survival. The impact on cost is inconclusive (low certainty). Breast cancer guidelines should be rigorously implemented in the clinical setting.

## Supplementary information

**Additional file 1.** Figure 1A. Breast cancer mortality in Europe 2018 - estimates. Figure 1B. Breast cancer incidence in Europe 2018 -estimates

**Additional file 2.** Search strategy

**Additional file 3.** Excluded studies with reasons for exclusion

**Additional file 4.** Characteristics of the included studies

**Additional file 5.** Adherence definitions and main findings

## Data Availability

The datasets used and/or analysed during the current study are available from the corresponding author on reasonable request.

## Abbreviations

CGs: Clinical guidelines
CI: Confidence interval
EU: European Union
HR: Hazard ratio
PRISMA: Preferred Reporting Items for Systematic Reviews and Meta-analysis
BRENDA: “Breast Cancer Care Under Evidence Based Guidelines” retrospective cohort project
TNBC: Triple Negative Breast Cancer
