# Supplementary material for: Adherence to breast cancer guidelines is associated with better survival outcomes: a systematic review and meta-analysis of observational studies in EU countries
Source: BMC Health Serv Res. 2020 Oct 7;20:920. doi: 10.1186/s12913-020-05753-x (PMC7542898; doi:10.1186/s12913-020-05753-x)
Supplement: Supplementary file 1 — Additional file 1. Figure 1A. Breast cancer mortality in Europe 2018 - estimates. Figure 1B. Breast cancer incidence in Europe 2018 -estimates [file 12913_2020_5753_MOESM1_ESM.docx]

**Additional file 1**

***Figure 1A. Breast cancer mortality in Europe 2018 - estimates***


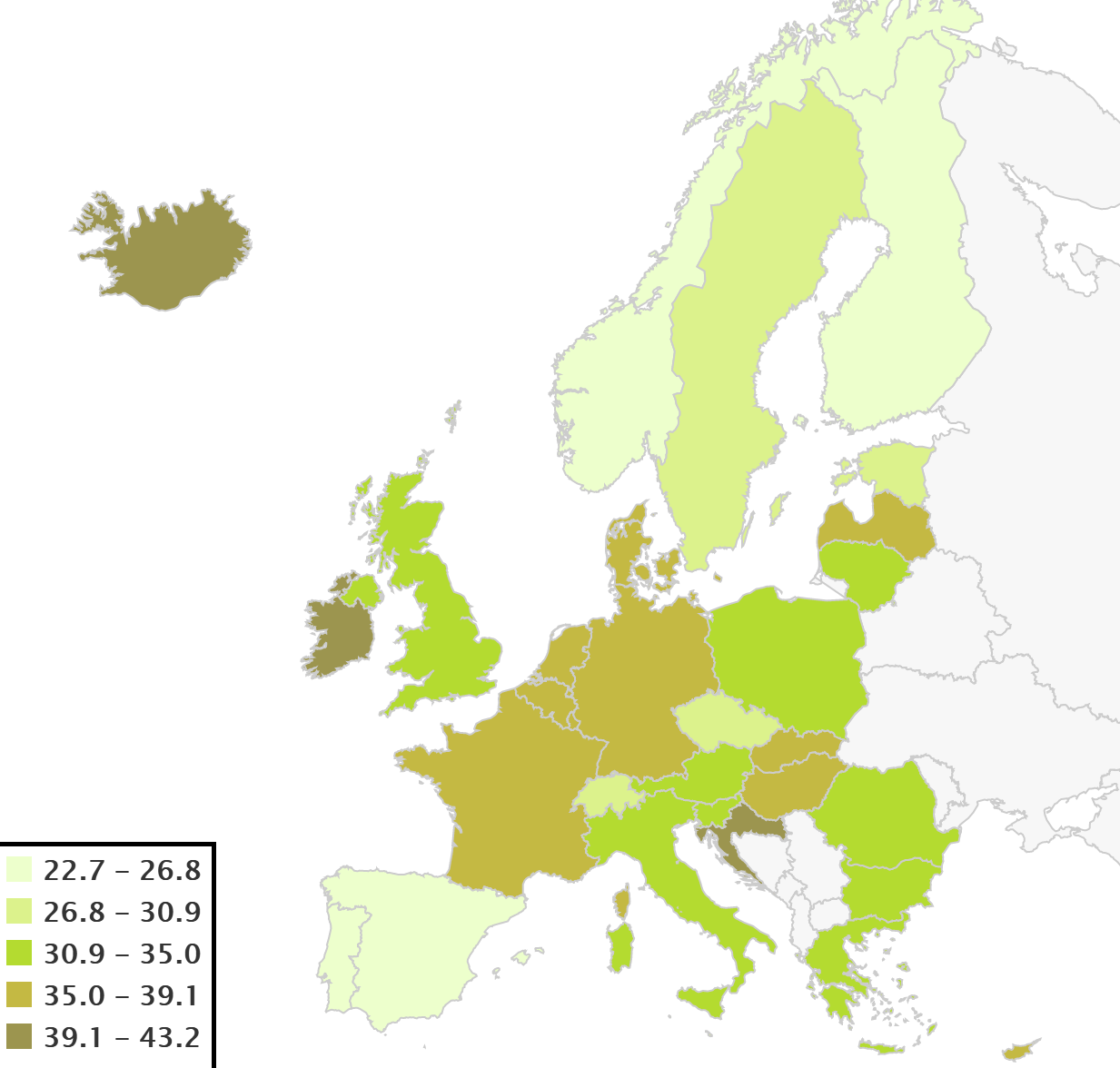


Age standardised breast cancer mortality rate per 100 000 (both sexes, all ages, 2018).

Source: ECIS - European Cancer Information System From https://ecis.jrc.ec.europa.eu, accessed on day/month/year © European Union, 2019

***Figure 1B. Breast cancer incidence in Europe 2018 -estimates***


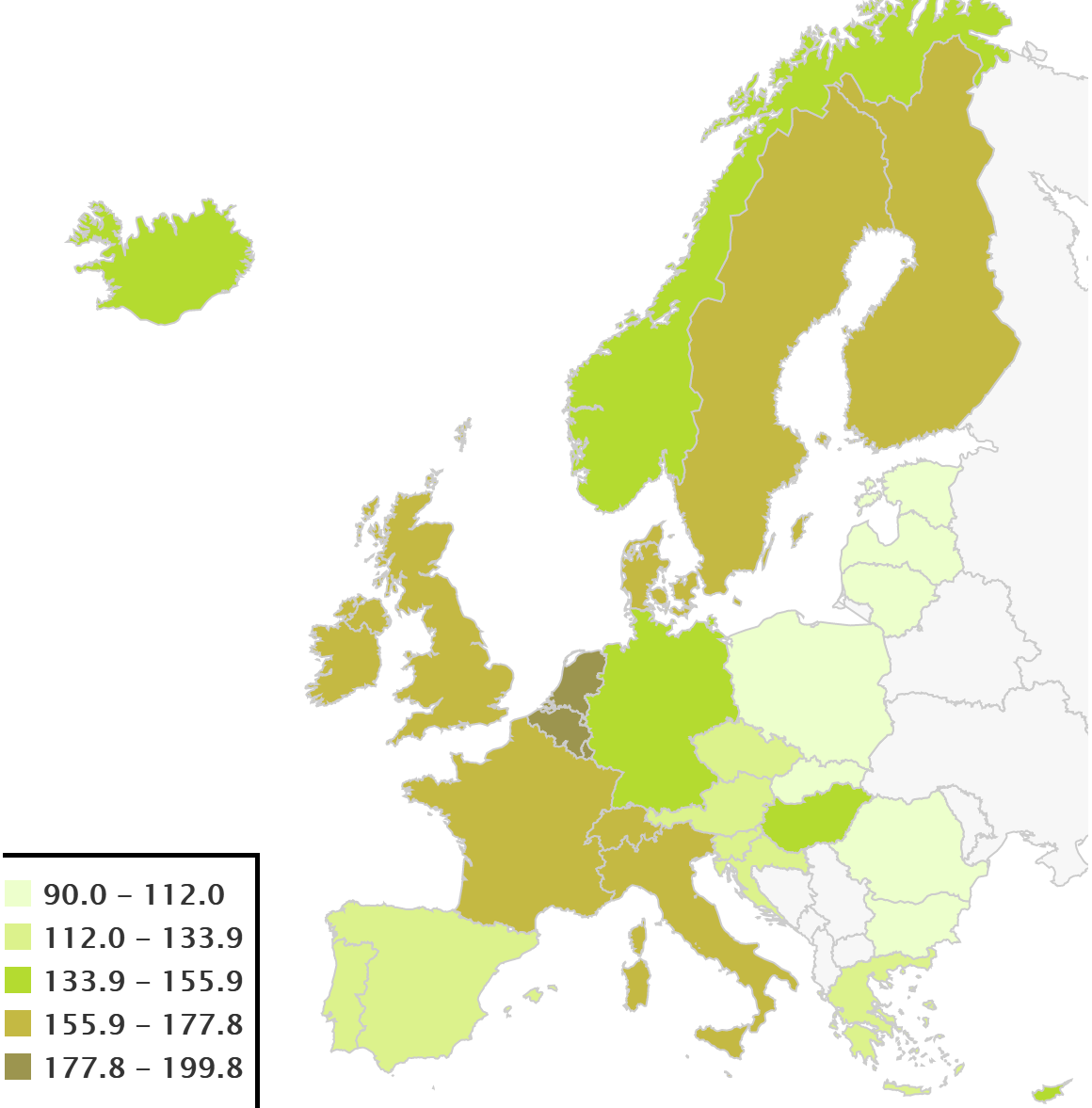


Age standardised breast cancer incidence rate per 100 000 (both sexes, all ages, 2018).

Source: ECIS - European Cancer Information System From https://ecis.jrc.ec.europa.eu, accessed on day/month/year © European Union, 2019
