# Supplementary material for: Adherence to breast cancer guidelines is associated with better survival outcomes: a systematic review and meta-analysis of observational studies in EU countries
Source: BMC Health Serv Res. 2020 Oct 7;20:920. doi: 10.1186/s12913-020-05753-x (PMC7542898; doi:10.1186/s12913-020-05753-x)
Supplement: Supplementary file 2 — Additional file 2. Search strategy [file 12913_2020_5753_MOESM2_ESM.docx]

**Additional file 2. Search strategy**

| **Systematic reviews** | |
| --- | --- |
| **MEDLINE**  PubMed  14/09/2017 | #1 Guideline Adherence”[Mesh]  #2 adherence[ti]  #3 implement*[ti]  #4 uptake[ti]  #5 complian*[ti]  #6 #1 OR #2 OR #3 OR #4 OR #5  #7 guideline*[tiab]  #8 recommendation*[ti]  #9 #7 OR #8  #10 #6 AND #9  #11 systematic[sb]  #12 framework*[tiab]  #13  #11 OR #12  #14 #10 AND #13 |
| **EMBASE**  Ovid Embase  <1980 to 2017 Week 38>  19/09/2017 | #1 adherence.ti.  #2 implement*.ti.  #3 uptake.ti.  #4 complian*.ti.  #5 #1 or #2 or #3 or #4  #6 guideline*.ti,ab.  #7 recommendation*.ti.  #8 #6 or# 7  #9 #5 and #8  #10 limit #9 to "systematic review"  #11 framework*.ti,ab.  #12 #9 and #11  #13 #10 or #12 |
| **Primary studies** | |
| **MEDLINE**  PubMed  14/09/2017 | #1 Guideline Adherence”[Mesh]  #2 adherence[ti]  #3 implement*[ti]  #4 uptake[ti  #5 complian*[ti]  #6 #1 OR #2 OR #3 OR #4 OR #5  #7 guideline*[tiab]  #8 recommendation*[ti]  #9 #7 OR #8  #10 #6 AND #9  #11 Neoplasms”[Majr]  #12 cancer[tiab]  #13 oncolog*[tiab]  #14 mammogram*[tiab  #15 breast[ti] AND screen*[ti]  #16 #11 OR #12 OR #13 OR #14 OR #15  #17  #10 AND #16 |
| **EMBASE**  Ovid Embase  <1980 to 2017 Week 38>  19/09/2017 | #1 adherence.ti.  #2  implement*.ti.  #3 uptake.ti.  #4 complian*.ti.  #5 #1 or# 2 or #3 or #4  #6 guideline*.ti,ab.  #7 recommendation*.ti.  #8  #6 or #7  #9  #5 and 8  #10 *neoplasm/  #11 cancer.ti,ab.  #12 oncolog*.ti,ab.  #13 mammogram*.ti,ab.  #14 (breast adj4 screen*).ti  #15 #10 or #11 or #12 or #13 or #14  #16 #9 and #15 |
