# Supplementary material for: Adherence to breast cancer guidelines is associated with better survival outcomes: a systematic review and meta-analysis of observational studies in EU countries
Source: BMC Health Serv Res. 2020 Oct 7;20:920. doi: 10.1186/s12913-020-05753-x (PMC7542898; doi:10.1186/s12913-020-05753-x)
Supplement: Supplementary file 3 — Additional file 3. Excluded studies with reasons for exclusion [file 12913_2020_5753_MOESM3_ESM.docx]

**Additional file 3. Excluded studies with reasons for exclusion**

| **Study ID** | **Reason for exclusion** |
| --- | --- |
| **Systematic reviews** | |
| 1. Gandhi, Verma et al. 2015(1) | Not focused on the impact of guideline adherence on health outcomes |
| 1. Henry, Hayes et al. 2014(2) | Not focused on the impact of guideline adherence on health outcomes |
| 1. Brouwers, De Vito et al. 2011(3) | Not focused on the impact of guideline adherence on health outcomes |
| 1. Gluck and Mamounas 2010(4) | Not focused on the impact of guideline adherence on health outcomes |
| 1. Eniu, Carlson et al. 2008(5) | Not focused on the impact of guideline adherence on health outcomes |
| 1. Harford, Azavedo et al. 2008(6) | Not focused on the impact of guideline adherence on health outcomes |
| 1. Sabatino, Habarta et al. 2008(7) | Not focused on the impact of guideline adherence on health outcomes |
| 1. Shyyan, Sener et al. 2008(8) | Not focused on the impact of guideline adherence on health outcomes |
| 1. Yip, Smith et al. 2008(9) | Not focused on the impact of guideline adherence on health outcomes |
| 1. Ackermann and Cheal 1994(10) | Not focused on the impact of guideline adherence on health outcomes |
| **Individual studies** |  |
| 1. Hamood, Hamood et al. 2019(11) | Not EU country |
| 1. Kim, Lee et al. 2019(12) | Not EU country |
| 1. Leinert, Schwentner et al. 2019(13) | No relevant outcome examined |
| 1. Lu and Spigelman 2019(14) | Not EU country |
| 1. Van Ryckeghem, Haverbeke et al. 2019(15) | No relevant outcome examined |
| 1. Watson, Deac et al. 2019(16) | No relevant outcome examined |
| 1. AlFarhan, Algwaiz et al. 2018(17) | No breast cancer |
| 1. Armson, Roder et al. 2018(18) | Adherence levels not measured |
| 1. Beaber, Sprague et al. 2018(19) | Not EU country |
| 1. DeSnyder, Hunt et al. 2018(20) | Not EU country |
| 1. Gilbo, Potters et al. 2018(21) | Adherence levels not measured |
| 1. Gray, Vandergrift et al. 2018(22) | Not EU country |
| 1. Hallowell, Puricelli Perin et al. 2018(23) | Not EU country |
| 1. Hill, Friend et al. 2018(24) | Not EU country |
| 1. Hill, Vang et al. 2018(25) | Adherence levels not measured |
| 1. Jensen, Laenkholm et al. 2018(26) | No relevant outcome examined |
| 1. Jones, Turton et al. 2018(27) | No relevant outcome examined |
| 1. Katz, Tengekyon et al. 2018(28) | Not EU country |
| 1. Luctkar-Flude, Aiken et al. 2018(29) | Not EU country |
| 1. Martel, Lambertini et al. 2018(30) | Not EU country |
| 1. Migowski, Dias et al. 2018(31) | Narrative review |
| 1. Mylvaganam, Conroy et al. 2018(32) | No relevant outcome examined |
| 1. Press, Ibraheem et al. 2018(33) | Not EU country |
| 1. Radhakrishnan, Nowak et al. 2018(34) | Not EU country |
| 1. Acuna, Sutradhar et al. 2017(35) | Not EU country |
| 1. Dull, Linkugel et al. 2017(36) | Not EU country |
| 1. Heins, de Jong et al. 2017(37) | No relevant outcome examined |
| 1. Heelan Gladden, et al. 2017 (38) |  |
| 1. Holm-Rasmussen, Jensen et al. 2017(39) | No relevant outcome examined |
| 1. Kuijer, Verloop et al. 2017(40) | No relevant outcome examined |
| 1. Patrick, Hasse et al. 2017(41) | Not EU country |
| 1. Schreuder, Kuijer et al. 2017(42) | No relevant outcome examined |
| 1. Kuijer A, Verloop et al 2017 (43) | No relevant outcome examined |
| 1. Boskovic, Gasparic et al. 2017(44) | No relevant outcome examined |
| 1. Stuber, van Ewijk et al. 2017(45) | No relevant outcome examined |
| 1. Schwentner, Van Ewijk et al. 2016(46) | No relevant outcome examined |
| 1. Smith, Side et al. 2016(47) | No relevant outcome examined |
| 1. Visser, van de Ven et al. 2016(48) | No relevant outcome examined |
| 1. Bouaud, Spano et al. 2015(49) | No relevant outcome examined |
| 1. Castello, Prieto et al. 2015(50) | Not focused on providers' adherence to CGs, but rather on patients' adherence to medical recommendations for breast cancer prevention |
| 1. Schrodi, Niedostatek et al. 2015)(51) | No relevant outcome examined |
| 1. Bouaud, Blaszka-Jaulerry et al. 2014(52) | No relevant outcome examined |
| 1. Eccher, Seyfang et al. 2014(53) | No relevant outcome examined |
| 1. Natoli, Brocco et al. 2014(54) | No relevant outcome examined |
| 1. Seroussi, Laouenan et al. 2013(55) | No relevant outcome examined |
| 1. Seroussi, Soulet et al. 2013(56) | No relevant outcome examined |
| 1. Grandjean, Kwast et al. 2012(57) | No relevant outcome examined |
| 1. Ray-Coquard, Morere et al. 2012(58) | No relevant outcome examined |
| 1. Seroussi, Soulet et al. 2012(59) | No relevant outcome examined |
| 1. Barni, Venturini et al. 2011(60) | No relevant outcome examined |
| 1. Bouaud and Seroussi 2011(61) | No relevant outcome examined |
| 1. De Munk et al. 2011 (62) | Focused on the impact of the implementation of the CGs |
| 1. Lebeau, Mathoulin-Pelissier et al. 2011(63) | No relevant outcome examined |
| 1. Liebrich, Unger et al. 2011(64) | No relevant outcome examined |
| 1. Saldanha, Garrett et al. 2011(65) | Adherence to CGs not examined |
| 1. Veerbeek, van der Geest et al. 2011(66) | No relevant outcome examined |
| 1. Weggelaar, Aben et al. 2011(67) | No relevant outcome examined |
| 1. Vercauteren, Kessels et al. 2010(68) | No relevant outcome examined |
| 1. Bucchi, Foca et al. 2009(69) | No relevant outcome examined |
| 1. Groot, Hommersom et al. 2009(70) | No relevant outcome examined |
| 1. Aristei, Amichetti et al. 2008(71) | No relevant outcome examined |
| 1. Jackisch, Untch et al. 2008(72) | Adherence levels not measured |
| 1. Hofvind, Geller et al. 2007(73) | Adherence of a screening programme to EU CGs, rather than healthcare providers' adherence to CGs |
| 1. Seroussi, Bouaud et al. 2007(74) | No relevant outcome examined |
| 1. Young, Valassiadou et al. 2007(75) | Adherence levels not measured |
| 1. Jensen, Mikkelsen et al. 2005(76) | Not focused on CGs, but rather on quality assurance CGs |
| 1. Schaapveld, de Vries et al. 2005(77) | No relevant outcome examined |
| 1. Schaapveld, de Vries et al. 2004(78) | No relevant outcome examined |
| 1. Balasubramanian, Murrow et al. 2003(79) | No relevant outcome examined |
| 1. Ottevanger et al. 2004 (80) | Focused on the impact of the implementation of the CGs, rather than on guldieline adherence |
| 1. DURTO 2003(81) | No relevant outcome examined |
| 1. Roila, Ballatori et al. 2003(82) | No relevant outcome examined |
| 1. Bouaud and Seroussi 2002(83) | No relevant outcome examined |
| 1. Palazzi, De Tomasi et al. 2002(84) | No relevant outcome examined |
| 1. Bouaud, Seroussi et al. 2001(85) | No relevant outcome examined |
| 1. Seroussi, Bouaud et al. 2001(86) | No relevant outcome examined |
| 1. Bell, Ma et al. 2000(87) | No relevant outcome examined |
| 1. Craft, Zhang et al. 2000(88) | Not EU country |
| 1. de Bock, Vliet Vlieland et al. 1999(89) | No relevant outcome examined |
| 1. Lane and Messina 1999(90) | Not EU country |
| 1. Rat-Coquard et al. 1997 (91) | Focused on the impact of the implementation of the CGs, rather than on CGs adherence |

**References (excluded papers)**

1. Gandhi S, Verma S, Ethier JL, Simmons C, Burnett H, Alibhai SM. A systematic review and quality appraisal of international guidelines for early breast cancer systemic therapy: Are recommendations sensitive to different global resources? Breast (Edinburgh, Scotland). 2015;24(4):309-17.

2. Henry NL, Hayes DF, Ramsey SD, Hortobagyi GN, Barlow WE, Gralow JR. Promoting quality and evidence-based care in early-stage breast cancer follow-up. Journal of the National Cancer Institute. 2014;106(4):dju034.

3. Brouwers MC, De Vito C, Bahirathan L, Carol A, Carroll JC, Cotterchio M, et al. What implementation interventions increase cancer screening rates? a systematic review. Implementation science : IS. 2011;6:111.

4. Gluck S, Mamounas T. Improving outcomes in early-stage breast cancer. Oncology (Williston Park, NY). 2010;24(11 Suppl 4):1-15.

5. Eniu A, Carlson RW, El Saghir NS, Bines J, Bese NS, Vorobiof D, et al. Guideline implementation for breast healthcare in low- and middle-income countries: treatment resource allocation. Cancer. 2008;113(8 Suppl):2269-81.

6. Harford J, Azavedo E, Fischietto M. Guideline implementation for breast healthcare in low- and middle-income countries: breast healthcare program resource allocation. Cancer. 2008;113(8 Suppl):2282-96.

7. Sabatino SA, Habarta N, Baron RC, Coates RJ, Rimer BK, Kerner J, et al. Interventions to increase recommendation and delivery of screening for breast, cervical, and colorectal cancers by healthcare providers systematic reviews of provider assessment and feedback and provider incentives. American journal of preventive medicine. 2008;35(1 Suppl):S67-74.

8. Shyyan R, Sener SF, Anderson BO, Garrote LM, Hortobagyi GN, Ibarra JA, Jr., et al. Guideline implementation for breast healthcare in low- and middle-income countries: diagnosis resource allocation. Cancer. 2008;113(8 Suppl):2257-68.

9. Yip CH, Smith RA, Anderson BO, Miller AB, Thomas DB, Ang ES, et al. Guideline implementation for breast healthcare in low- and middle-income countries: early detection resource allocation. Cancer. 2008;113(8 Suppl):2244-56.

10. Ackermann SP, Cheal N. Factors affecting physician adherence to breast cancer screening guidelines. Journal of cancer education : the official journal of the American Association for Cancer Education. 1994;9(2):96-100.

11. Hamood R, Hamood H, Merhasin I, Keinan-Boker L. Hormone therapy and osteoporosis in breast cancer survivors: assessment of risk and adherence to screening recommendations. Osteoporosis International. 2019;30(1):187-200.

12. Kim HK, Lee SH, Kim YJ, Park SE, Lee HS, Lim SW, et al. Does guideline non-adherence result in worse clinical outcomes for hormone receptor-positive and HER2-negative metastatic breast cancer in premenopausal women?: Result of an institution database from South Korea 11 Medical and Health Sciences 1112 Oncology and Carcinogenesis. BMC Cancer. 2019;19(1):84.

13. Leinert E, Schwentner L, Blettner M, Wockel A, Felberbaum R, Flock F, et al. Association between cognitive impairment and guideline adherence for application of chemotherapy in older patients with breast cancer: Results from the prospective multicenter BRENDA II study. Breast Journal. 2019.

14. Lu M, Spigelman AD. Adherence to referral guidelines: Genetic testing in an Australian triple negative breast cancer (TNBC) cohort. International Journal of Health Governance. 2019;24(1):6-18.

15. Van Ryckeghem F, Haverbeke C, Wynendaele W, Jerusalem G, Somers L, Van den Broeck A, et al. Real-world use of granulocyte colony-stimulating factor in ambulatory breast cancer patients: a cross-sectional study. Supportive care in cancer : official journal of the Multinational Association of Supportive Care in Cancer. 2019;27(3):1099-108.

16. Watson GA, Deac O, Aslam R, O'Dwyer R, Tierney A, Sukor S, et al. Real-World Experience of Palbociclib-Induced Adverse Events and Compliance With Complete Blood Count Monitoring in Women With Hormone Receptor-Positive/HER2-Negative Metastatic Breast Cancer. Clinical Breast Cancer. 2019;19(1):e186-e94.

17. AlFarhan HA, Algwaiz GF, Alzahrani HA, Alsuhaibani RS, Alolayan A, Abdelhafiz N, et al. Impact of GI tumor board on patient management and adherence to guidelines. Journal of Global Oncology. 2018;2018(4).

18. Armson H, Roder S, Elmslie T, Khan S, Straus SE. How do clinicians use implementation tools to apply breast cancer screening guidelines to practice? Implementation science : IS. 2018;13(1):79.

19. Beaber EF, Sprague BL, Tosteson ANA, Haas JS, Onega T, Schapira MM, et al. Multilevel Predictors of Continued Adherence to Breast Cancer Screening Among Women Ages 50-74 Years in a Screening Population. Journal of women's health (2002). 2018.

20. DeSnyder SM, Hunt KK, Dong W, Smith BD, Moran MS, Chavez-MacGregor M, et al. American Society of Breast Surgeons' Practice Patterns After Publication of the SSO-ASTRO-ASCO DCIS Consensus Guideline on Margins for Breast-Conserving Surgery With Whole-Breast Irradiation. Ann Surg Oncol. 2018;25(10):2965-74.

21. Gilbo P, Potters L, Lee L. Implementation and utilization of hypofractionation for breast cancer. Advances in Radiation Oncology. 2018;3(3):265-70.

22. Gray BM, Vandergrift JL, Lipner RS. Association between the American Board of Internal Medicine's General Internist's Maintenance of Certification Requirement and Mammography Screening for Medicare Beneficiaries. Women's health issues : official publication of the Jacobs Institute of Women's Health. 2018;28(1):35-41.

23. Hallowell BD, Puricelli Perin DM, Simoes EJ, Paez DC, Parra DC, Brownson RC, et al. Breast cancer related perceptions and practices of health professionals working in Brazil's network of primary care units. Prev Med. 2018;106:216-23.

24. Hill DA, Friend S, Lomo L, Wiggins C, Barry M, Prossnitz E, et al. Breast cancer survival, survival disparities, and guideline-based treatment. Breast Cancer Res Treat. 2018;170(2):405-14.

25. Hill LA, Vang CA, Kennedy CR, Linebarger JH, Dietrich LL, Parsons BM, et al. A strategy for changing adherence to national guidelines for decreasing laboratory testing for early breast cancer patients. Wisconsin Medical Journal. 2018;117(2):68-72.

26. Jensen MB, Laenkholm AV, Offersen BV, Christiansen P, Kroman N, Mouridsen HT, et al. The clinical database and implementation of treatment guidelines by the Danish Breast Cancer Cooperative Group in 2007-2016. Acta Oncologica. 2018;57(1):13-8.

27. Jones S, Turton P, Almerie Q, Aldoori J, Achuthan R. Management and surveillance of women diagnosed with breast cancer with a family history of breast cancer: Are we nice compliant? European Journal of Surgical Oncology. 2018;44(6):903.

28. Katz D, Tengekyon AJ, Kahan NR, Calderon-Margalit R. Patient and physician characteristics affect adherence to screening mammography: A population-based cohort study. PLoS ONE. 2018;13(3):e0194409.

29. Luctkar-Flude M, Aiken A, McColl MA, Tranmer J. What do primary care providers think about implementing breast cancer survivorship care? Current Oncology. 2018;25(3):196-205.

30. Martel S, Lambertini M, Simon R, Matte C, Prady C. Adherence to guidelines in requesting oncotype DX in a publicly funded health care system. Current Oncology. 2018;25(4):e311-e8.

31. Migowski A, Dias MBK, Nadanovsky P, Silva GAE, Sant'Ana DR, Stein AT. Guidelines for early detection of breast cancer in Brazil. III - Challenges for implementation. Cad Saude Publica. 2018;34(6):e00046317.

32. Mylvaganam S, Conroy EJ, Williamson PR, Barnes NLP, Cutress RI, Gardiner MD, et al. Adherence to best practice consensus guidelines for implant-based breast reconstruction: Results from the iBRA national practice questionnaire survey. European journal of surgical oncology : the journal of the European Society of Surgical Oncology and the British Association of Surgical Oncology. 2018;44(5):708-16.

33. Press DJ, Ibraheem A, Dolan ME, Goss KH, Conzen S, Huo D. Racial disparities in omission of oncotype DX but no racial disparities in chemotherapy receipt following completed oncotype DX test results. Breast Cancer Res Treat. 2018;168(1):207-20.

34. Radhakrishnan A, Nowak SA, Parker AM, Visvanathan K, Pollack CE. Linking physician attitudes to their breast cancer screening practices: A survey of US primary care providers and gynecologists. Prev Med. 2018;107:90-102.

35. Acuna SA, Sutradhar R, Camacho X, Daly C, Del Giudice ME, Kim SJ, et al. Uptake of Cancer Screening Tests Among Recipients of Solid Organ Transplantation. American journal of transplantation : official journal of the American Society of Transplantation and the American Society of Transplant Surgeons. 2017;17(9):2434-43.

36. Dull B, Linkugel A, Margenthaler JA, Cyr AE. Overuse of chest CT in patients with stage I & II breast cancer: An opportunity to increase guidelines compliance at an NCCN member institution. JNCCN Journal of the National Comprehensive Cancer Network. 2017;15(6):783-9.

37. Heins MJ, de Jong JD, Spronk I, Ho VKY, Brink M, Korevaar JC. Adherence to cancer treatment guidelines: influence of general and cancer-specific guideline characteristics. European journal of public health. 2017;27(4):616-20.

38. Heelan Gladden AA, Sams S, Gleisner A, Finlayson C, Kounalakis N, Hosokawa P, et al. Re-excision rates after breast conserving surgery following the 2014 SSO-ASTRO guidelines. American journal of surgery. 2017;214(6):1104-9.

39. Holm-Rasmussen EV, Jensen MB, Balslev E, Kroman N, Tvedskov TF. The use of sentinel lymph node biopsy in the treatment of breast ductal carcinoma in situ: A Danish population-based study. European journal of cancer (Oxford, England : 1990). 2017;87:1-9.

40. Kuijer A, Verloop J, Visser O, Sonke G, Jager A, van Gils CH, et al. The influence of socioeconomic status and ethnicity on adjuvant systemic treatment guideline adherence for early-stage breast cancer in the Netherlands. Annals of Oncology. 2017;28(8):1970-8.

41. Patrick JL, Hasse ME, Feinglass J, Khan SA. Trends in adherence to NCCN guidelines for breast conserving therapy in women with Stage I and II breast cancer: Analysis of the 1998-2008 National Cancer Data Base. Surgical Oncology. 2017;26(4):359-67.

42. Schreuder K, Kuijer A, Rutgers EJT, Smorenburg CH, van Dalen T, Siesling S. Impact of gene-expression profiling in patients with early breast cancer when applied outside the guideline directed indication area. European journal of cancer (Oxford, England : 1990). 2017;84:270-7.

43. Kuijer A, Verloop J, Visser O, Sonke G, Jager A, van Gils CH, et al. The influence of socioeconomic status and ethnicity on adjuvant systemic treatment guideline adherence for early-stage breast cancer in the Netherlands. Annals of oncology : official journal of the European Society for Medical Oncology. 2017;28(8):1970-8.

44. Boskovic L, Gasparic M, Petkovic M, Gugic D, Lovasic IB, Soldic Z, et al. Bone health and adherence to vitamin D and calcium therapy in early breast cancer patients on endocrine therapy with aromatase inhibitors. Breast (Edinburgh, Scotland). 2017;31:16-9.

45. Stuber T, van Ewijk R, Diessner J, Kuhn T, Flock F, Felberbaum R, et al. Which patient- and physician-related factors are associated with guideline adherent initiation of adjuvant endocrine therapy? Results of the prospective multi-centre cohort study BRENDA II. Breast cancer (Tokyo, Japan). 2017;24(2):281-7.

46. Schwentner L, Van Ewijk R, Kuhn T, Flock F, Felberbaum R, Blettner M, et al. Exploring patient- and physician-related factors preventing breast cancer patients from guideline-adherent adjuvant chemotherapy-results from the prospective multi-center study BRENDA II. Supportive care in cancer : official journal of the Multinational Association of Supportive Care in Cancer. 2016;24(6):2759-66.

47. Smith SG, Side L, Meisel SF, Horne R, Cuzick J, Wardle J. Clinician-Reported Barriers to Implementing Breast Cancer Chemoprevention in the UK: A Qualitative Investigation. Public health genomics. 2016;19(4):239-49.

48. Visser A, van de Ven EM, Ruczynski LI, Blaisse RJ, van Halteren HK, Aben K, et al. Cardiac monitoring during adjuvant trastuzumab therapy: Guideline adherence in clinical practice. Acta oncologica (Stockholm, Sweden). 2016;55(4):423-9.

49. Bouaud J, Spano JP, Lefranc JP, Cojean-Zelek I, Blaszka-Jaulerry B, Zelek L, et al. Physicians' Attitudes Towards the Advice of a Guideline-Based Decision Support System: A Case Study With OncoDoc2 in the Management of Breast Cancer Patients. Studies in health technology and informatics. 2015;216:264-9.

50. Castello A, Prieto L, Ederra M, Salas-Trejo D, Vidal C, Sanchez-Contador C, et al. Association between the Adherence to the International Guidelines for Cancer Prevention and Mammographic Density. PloS one. 2015;10(7):e0132684.

51. Schrodi S, Niedostatek A, Werner C, Tillack A, Schubert-Fritschle G, Engel J. Is primary surgery of breast cancer patients consistent with German guidelines? Twelve-year trend of population-based clinical cancer registry data. European journal of cancer care. 2015;24(2):242-52.

52. Bouaud J, Blaszka-Jaulerry B, Zelek L, Spano JP, Lefranc JP, Cojean-Zelek I, et al. Health information technology: use it well, or don't! Findings from the use of a decision support system for breast cancer management. AMIA Annual Symposium proceedings AMIA Symposium. 2014;2014:315-24.

53. Eccher C, Seyfang A, Ferro A. Implementation and evaluation of an Asbru-based decision support system for adjuvant treatment in breast cancer. Computer methods and programs in biomedicine. 2014;117(2):308-21.

54. Natoli C, Brocco D, Sperduti I, Nuzzo A, Tinari N, De Tursi M, et al. Breast cancer "tailored follow-up" in Italian oncology units: a web-based survey. PloS one. 2014;9(4):e94063.

55. Seroussi B, Laouenan C, Gligorov J, Uzan S, Mentre F, Bouaud J. Which breast cancer decisions remain non-compliant with guidelines despite the use of computerised decision support? British journal of cancer. 2013;109(5):1147-56.

56. Seroussi B, Soulet A, Spano JP, Lefranc JP, Cojean-Zelek I, Blaszka-Jaulerry B, et al. Which patients may benefit from the use of a decision support system to improve compliance of physician decisions with clinical practice guidelines: a case study with breast cancer involving data mining. Studies in health technology and informatics. 2013;192:534-8.

57. Grandjean I, Kwast AB, de Vries H, Klaase J, Schoevers WJ, Siesling S. Evaluation of the adherence to follow-up care guidelines for women with breast cancer. European journal of oncology nursing : the official journal of European Oncology Nursing Society. 2012;16(3):281-5.

58. Ray-Coquard I, Morere JF, Scotte F, Cals L, Antoine EC. Management of anemia in advanced breast and lung cancer patients in daily practice: results of a French survey. Advances in therapy. 2012;29(2):124-33.

59. Seroussi B, Soulet A, Messai N, Laouenan C, Mentre F, Bouaud J. Patient clinical profiles associated with physician non-compliance despite the use of a guideline-based decision support system: a case study with OncoDoc2 using data mining techniques. AMIA Annual Symposium proceedings AMIA Symposium. 2012;2012:828-37.

60. Barni S, Venturini M, Molino A, Donadio M, Rizzoli S, Maiello E, et al. Importance of adherence to guidelines in breast cancer clinical practice. The Italian experience (AIOM). Tumori. 2011;97(5):559-63.

61. Bouaud J, Seroussi B. Revisiting the EBM decision model to formalize non-compliance with computerized CPGs: results in the management of breast cancer with OncoDoc2. AMIA Annual Symposium proceedings AMIA Symposium. 2011;2011:125-34.

62. de Munck L, Schaapveld M, Siesling S, Wesseling J, Voogd AC, Tjan-Heijnen VC, et al. Implementation of trastuzumab in conjunction with adjuvant chemotherapy in the treatment of non-metastatic breast cancer in the Netherlands. Breast cancer research and treatment. 2011;129(1):229-33.

63. Lebeau M, Mathoulin-Pelissier S, Bellera C, Tunon-de-Lara C, Daban A, Lipinski F, et al. Breast cancer care compared with clinical Guidelines: an observational study in France. BMC public health. 2011;11:45.

64. Liebrich C, Unger G, Dlugosch B, Hofmann S, Petry KU. Adopting Guidelines into Clinical Practice: Implementation of Trastuzumab in the Adjuvant Treatment of Breast Cancer in Lower Saxony, Germany, in 2007. Breast care (Basel, Switzerland). 2011;6(1):43-50.

65. Saldanha JD, Garrett RM, Snaddon L, Longmuir M, Bradshaw N, Watt C, et al. Impact of national guidelines on family history breast cancer surveillance. Scottish medical journal. 2011;56(4):203-5.

66. Veerbeek L, van der Geest L, Wouters M, Guicherit O, Does-den Heijer A, Nortier J, et al. Enhancing the quality of care for patients with breast cancer: seven years of experience with a Dutch auditing system. European journal of surgical oncology : the journal of the European Society of Surgical Oncology and the British Association of Surgical Oncology. 2011;37(8):714-8.

67. Weggelaar I, Aben KK, Warle MC, Strobbe LJ, van Spronsen DJ. Declined guideline adherence in older breast cancer patients: a population-based study in the Netherlands. The breast journal. 2011;17(3):239-45.

68. Vercauteren LD, Kessels AG, van der Weijden T, Severens JL, van Engelshoven JM, Flobbe K. Association between guideline adherence and clinical outcome for patients referred for diagnostic breast imaging. Quality & safety in health care. 2010;19(6):503-8.

69. Bucchi L, Foca F, Ravaioli A, Vattiato R, Balducci C, Fabbri C, et al. Receipt of adjuvant systemic therapy among patients with high-risk breast cancer detected by mammography screening. Breast cancer research and treatment. 2009;113(3):559-66.

70. Groot P, Hommersom A, Lucas PJ, Merk RJ, ten Teije A, van Harmelen F, et al. Using model checking for critiquing based on clinical guidelines. Artificial intelligence in medicine. 2009;46(1):19-36.

71. Aristei C, Amichetti M, Ciocca M, Nardone L, Bertoni F, Vidali C. Radiotherapy in Italy after conservative treatment of early breast cancer. A survey by the Italian Society of Radiation Oncology (AIRO). Tumori. 2008;94(3):333-41.

72. Jackisch C, Untch M, Chatsiproios D, Lamparter C, Overkamp F, Lichtenegger W, et al. Adherence to Treatment Guidelines in Breast Cancer Care - a Retrospective Analysis of the 'Organgruppe Mamma der Arbeitsgemeinschaft Gynaekologische Onkologie'. Breast care (Basel, Switzerland). 2008;3(2):87-92.

73. Hofvind S, Geller B, Vacek PM, Thoresen S, Skaane P. Using the European guidelines to evaluate the Norwegian Breast Cancer Screening Program. European journal of epidemiology. 2007;22(7):447-55.

74. Seroussi B, Bouaud J, Gligorov J, Uzan S. Supporting multidisciplinary staff meetings for guideline-based breast cancer management: a study with OncoDoc2. AMIA Annual Symposium proceedings AMIA Symposium. 2007:656-60.

75. Young OE, Valassiadou K, Dixon M. A review of current practices in breast conservation surgery in the UK. Annals of the Royal College of Surgeons of England. 2007;89(2):118-23.

76. Jensen A, Mikkelsen GJ, Vestergaard M, Lynge E, Vejborg I. Compliance with European guidelines for diagnostic mammography in a decentralized health-care setting. Acta radiologica (Stockholm, Sweden : 1987). 2005;46(2):140-7.

77. Schaapveld M, de Vries EG, Otter R, de Vries J, Dolsma WV, Willemse PH. Guideline adherence for early breast cancer before and after introduction of the sentinel node biopsy. British journal of cancer. 2005;93(5):520-8.

78. Schaapveld M, de Vries EG, van der Graaf WT, Otter R, Willemse PH. Quality of adjuvant CMF chemotherapy for node-positive primary breast cancer: a population-based study. Journal of cancer research and clinical oncology. 2004;130(10):581-90.

79. Balasubramanian SP, Murrow S, Holt S, Manifold IH, Reed MW. Audit of compliance to adjuvant chemotherapy and radiotherapy guidelines in breast cancer in a cancer network. Breast (Edinburgh, Scotland). 2003;12(2):136-41.

80. Ottevanger PB, De Mulder PH, Grol RP, van Lier H, Beex LV. Adherence to the guidelines of the CCCE in the treatment of node-positive breast cancer patients. European journal of cancer (Oxford, England : 1990). 2004;40(2):198-204.

81. DURTO. Antiemetic prescription in Italian breast cancer patients submitted to adjuvant chemotherapy. Supportive care in cancer : official journal of the Multinational Association of Supportive Care in Cancer. 2003;11(12):785-9.

82. Roila F, Ballatori E, Patoia L, Palazzo S, Veronesi A, Frassoldati A, et al. Adjuvant systemic therapies in women with breast cancer: an audit of clinical practice in Italy. Annals of oncology : official journal of the European Society for Medical Oncology. 2003;14(6):843-8.

83. Bouaud J, Seroussi B. Impact of site-specific customizations on physician compliance with guidelines. Studies in health technology and informatics. 2002;90:543-7.

84. Palazzi M, De Tomasi D, D'Affronto C, Richetti A, Valli MC, Meregalli S, et al. Are international guidelines for the prescription of adjuvant treatment for early breast cancer followed in clinical practice? Results of a population-based study on 1547 patients. Tumori. 2002;88(6):503-6.

85. Bouaud J, Seroussi B, Antoine EC, Zelek L, Spielmann M. A before-after study using OncoDoc, a guideline-based decision support-system on breast cancer management: impact upon physician prescribing behaviour. Studies in health technology and informatics. 2001;84(Pt 1):420-4.

86. Seroussi B, Bouaud J, Antoine EC. ONCODOC: a successful experiment of computer-supported guideline development and implementation in the treatment of breast cancer. Artificial intelligence in medicine. 2001;22(1):43-64.

87. Bell CM, Ma M, Campbell S, Basnett I, Pollock A, Taylor I. Methodological issues in the use of guidelines and audit to improve clinical effectiveness in breast cancer in one United Kingdom health region. European journal of surgical oncology : the journal of the European Society of Surgical Oncology and the British Association of Surgical Oncology. 2000;26(2):130-6.

88. Craft PS, Zhang Y, Brogan J, Tait N, Buckingham JM. Implementing clinical practice guidelines: A community-based audit of breast cancer treatment. Medical Journal of Australia. 2000;172(5):213-6.

89. de Bock GH, Vliet Vlieland TP, Hakkeling M, Kievit J, Springer MP. GPs' management of women seeking help for familial breast cancer. Family practice. 1999;16(5):463-7.

90. Lane DS, Messina CR. Methodology for targeting physicians for interventions to improve breast cancer screening. American journal of preventive medicine. 1999;16(4):289-97.

91. Ray-Coquard I, Philip T, Lehmann M, Fervers B, Farsi F, Chauvin F. Impact of a clinical guidelines program for breast and colon cancer in a French cancer center. Jama. 1997;278(19):1591-5.
