# Supplementary material for: Adherence to breast cancer guidelines is associated with better survival outcomes: a systematic review and meta-analysis of observational studies in EU countries
Source: BMC Health Serv Res. 2020 Oct 7;20:920. doi: 10.1186/s12913-020-05753-x (PMC7542898; doi:10.1186/s12913-020-05753-x)
Supplement: Supplementary file 4 — Additional file 4. Characteristics of the included studies [file 12913_2020_5753_MOESM4_ESM.docx]

**Additional file 4. Characteristics of included studies**

| **Author(s)/ publication year** | **Country** | **Aim** | **Year of study** | **CGs scope** | **n** | **Patient’s characteristics** | **Outcomes** | **Risk of Bias** |
| --- | --- | --- | --- | --- | --- | --- | --- | --- |
| **Non-controlled before-after study** | | | | | | | | |
| Mille, Roy et al. 2000 ([31](#_ENREF_31)) | France | To evaluate the economic impact of changing medical practices, which are evolving toward CG compliance, in the post-therapeutic follow-up of patients with localised breast cancer. | 1993, 1995. | Follow-up | 200 | Patient records were chosen at random for the analysis | Costs associated to adherence to CG about recommendations for patient follow ups | Moderate |
| Sacerdote, Bordon et al 2013 ([33](#_ENREF_33)) | Italy | To evaluate the impact of regional CGs (Piedmont CGs, PGL) for breast cancer diagnosis and treatment on quality-of-care indicators in the North-western Italian region of Piedmont. | 2002,2004 | Diagnosis and treatment | 600 and  621 | Women aged 50–69 years with incident breast cancer before and after the Piedmont CGs (PGL): | Overall survival | Low |
| Jacke, Albert et al 2015 ([17](#_ENREF_17)) | Germany | To assess guideline adherence according to time intervals and its impact on survival | 1996 - 1997, and 2003 - 2004 | Treatment | 877 | All women with primary BC treatment in two general hospitals and one specialised academic hospital located in the district of Marburg-Biedenkopf (Hesse, Germany) were included (entry cohort). | Overall survival | Low |
| **Retrospective cohort studies** | | | | | | | | |
| Andreano, Rebora et al. 2017([13](#_ENREF_13)) | Italy | To investigate the effect of adherence to CGs on long-term survival for a cohort of Italian breast cancer patients. | 2007–2012 | Diagnosis and treatment | 6 333 | Incident female breast cancer cases, not metastatic at diagnosis and receiving primary surgery. from the registry of the Milan province (Italy) | Overall survival | Low |
| de Roos et al. ([29](#_ENREF_29)) | Netherlands | To study the effect of compliance with CGs on local recurrence (LR)-free survival in patients treated for ductal carcinoma in situ (DCIS). | 1992–1995, 1996–1999 and 2000–2003 | Treatment | 251 | Consecutive patients treated for DCIS University of Groningen Medical Centre and the Martini Hospital. | Recurrence-free survival | Low |
| Ebner, Hancke et al 2015 ([14](#_ENREF_14)) | Germany | To investigate the associations among tumour characteristics, guideline adherence, and outcomes and to compare these associations between younger breast cancer patients (age 50-69 years) and older breast cancer patients (age≥ 70 years). | 1992 - 2008 | Treatment | 7 732 | Patients aged 50 or more with a diagnosis of a histologically confirmed invasive breast cancer (BRENDA cohort) | Overall survival  Disease-free survival | Moderate |
| Ebner, van Ewijk et al2015([15](#_ENREF_15)) | Germany | To investigate correlations between the biological subgroups, age, and guideline-adherent treatment, and examine the extent to which non-guideline-adherent-treatment concerning radiotherapy, surgery, endocrine-therapy and chemotherapy are associated with survival among the four biological subgroups of younger and older breast cancer patients. | 1992 - 2008 | Treatment | 5 632 | Patients with primary breast cancer diagnosed and treated in the Department of Gynaecology and Obstetrics of the University of Ulm and the surrounding 16 hospitals (BRENDA cohort) | Overall survival  Disease-free survival | Moderate |
| Hancke, Denkinger et al. 2010([30](#_ENREF_30)) | Germany | To examine the extent to which non-adherence to treatment CGs occurs for women aged ≥70 years and changes overall survival (OS) and disease-free survival (DFS). | 1992 - 2005 | Treatment | 1 922 | Women aged 50 or more with histologically confirmed invasive breast cancer University of Ulm .(BRENDA cohort) | Overall survival  Disease free survival | Low |
| Poncet, Colin et al. 2009 ([32](#_ENREF_32)) | France | To analyse adherence to prescribing CGs of anti-HER2 monoclonal antibody trastuzumab treatment for metastatic breast cancer. Efficacy and costs were also evaluated. | 1999-2003 | Treatment | 131 | Patients with metastatic breast cancer receiving trastuzumab treatment in four French healthcare centres. | Objective response rate  Overall survival  Progression-free survival  Cost | Low |
| Schwentner, Wolters et al. 2012(a) ([35](#_ENREF_35)) | Germany | To examine treatment patterns and the influence of guideline adherence on the survival of patients with bilateral breast cancer. To identify those guideline violations of adjuvant therapy (chemotherapy, radiotherapy, operation regime, endocrine therapy), which have the most important impact on RFS and OS. | 1992 -2008 | Treatment | 5 292 | Patients with primary breast cancer diagnosed or treated Department of Gynaecology and Obstetrics (University of Ulm) and 16 partner clinics (all certified breast cancer centres) in Baden Württemberg (Germany, BRENDA cohort | Overall survival  Recurrence-free surviva (RFS)l | Low |
| Schwentner, Wolters et al. 2012 (b) ([36](#_ENREF_36)) | Germany | To investigate the following issues: (1) Is there an impact of guideline-adherent treatment on RFS and OS in TNBC? (2) Which adjuvant treatment has the most important impact on RFS and OS in TNBC? | 2000 - 2005 | Treatment | 3 658 | Patients with primary breast cancer diagnosed or treated at the Department of Gynaecology and Obstetrics at the University of Ulm and 16 partner clinics in Baden-Württemberg (Germany) BRENDA cohort | Overall survival  Disease-free survival | Low |
| Schwentner, Wockel et al 2013([34](#_ENREF_34)) | Germany | To evaluate possible differences in survival in triple-negative breast cancer patients by age and by the extent of adherence to evidence-based treatment CGs | 1992 -2008 | Treatment | 9 156 | Patients with primary breast cancer diagnosed or treated at the Department of Gynaecology and Obstetrics at the University of Ulm and 16 partner clinics in Baden-Württemberg (Germany, BRENDA cohort ) | Overall survival  Disease free survival | Low |
| van de Water, Bastiaannet et al. 2012 ([37](#_ENREF_37)) | Netherlands | To assess adherence to treatment CGs by age at diagnosis, and to examine age-specific survival in relation to adherence to CGs | 2005 - 2008 | Treatment | 31 520 | Women with early-stage breast cancer (The Netherlands Cancer Registry database) | Overall survival | Low |
| Van Ewijk , Wockel et al 2015 ([38](#_ENREF_38)) | Germany | To examine the extent to which guideline-adherent adjuvant treatment is an equal alternative for elderly patients aged 65–80 | 1992 -2008 | Treatment | 4 142 | Patients with primary breast cancer in 17 participating breast cancer centres.(BRENDA cohort) | Overall survival  Recurrence free survival | Moderate |
| Varga, Wischnewsky et al. 2010 ([39](#_ENREF_39)) | Germany | To evaluate the association between guideline-adherent versus non-adherent treatment on recurrence-free survival (RFS) and overall survival (OS) in early-onset breast cancer patients | 1992-2005 | Treatment | 1 778 | 35 years or younger: 111  36 - 55 years: 1,667 .(BRENDA cohort) | Overall survival  Recurrence-free survival | Low |
| Wimmer Theresa et al.  2019([43](#_ENREF_43)) | Germany | To assess adherence to guidelines, long-term survival, recurrence rates, and recurrence-free survival after adjuvant radiotherapy (RT) in patients with breast-conserving therapy (BCT) in daily clinical practice. | 2003-2013 | Treatment | 6 370 | Patients with invasive non-metastatic breast cancer of tumour stage I, II, and III and breast-conserving therapy. Mean age of 59.5 years. | Long-term survival  Recurrence rate  Recurrence-free survival | Low |
| Wockel, Varga et al. 2010 ([40](#_ENREF_40)) | Germany | To investigate the influence of the national guideline on outcomes for breast cancer patients, including data from a period of 13 years | 1992 – 2005 | Treatment | 2 231 | Patients with primary breast cancer treated at the Department of Obstetrics and Gynaecology, University of Ulm, Germany (BRENDA cohort) | Overall survival  Recurrence-free survival | Low |
| Wockel, Wolters et al. 2014 ([42](#_ENREF_42)) | Germany | To answer the following questions in a daily routine cohort of breast cancer patients:  Does guideline-adherent radiotherapy (RT) improve primary breast cancer patient survival?  Is breast-conserving surgery (BCS) followed by RT equal to a mastectomy with regard to outcome parameters?  Does adjuvant RT compensate for an incomplete tumour resection (R1)? | 1992 - 2008 | Treatment | 8 935 | Primary breast cancer patients recruited from 17 participating certified breast cancer centres in Germany(BRENDA cohort) | Overall survival  Recurrence-free survival | Low |
| Wockel, Kurzeder et al. 2010 ([41](#_ENREF_41)) | Germany | To analyse the impact of German-S3-breast cancer guideline adherence on clinical outcomes. | 2001 - 2005 | Treatment | 3 976 | Patients with primary breast cancer treated in the Department of Obstetrics and Gynaecology, University of Ulm Medical Centre or local affiliated hospitals certified as breast centres(BRENDA cohort) | Overall survival  Recurrence free survival | Low |
| Wollschlager, Meng et al. 2017([16](#_ENREF_16)) | Germany | To investigate the association between baseline comorbidity, age, and the administration of surgery as well as three different adjuvant treatment modalities (chemotherapy, radiotherapy, and endocrine therapy).  To investigate the impact of guideline-adherent adjuvant treatment on survival in comorbid patients defined by the Charlson comorbidity index. | 1992 - 2008 | Treatment | 2 137 | Women with primary breast cancer diagnosed or treated Germany at the Department of Gynaecology and Obstetrics at the University of Ulm (BRENDA cohort). | Overall survival  Disease-free survival | Low |
| Wolters, Wischhusen et al. 2015 ([18](#_ENREF_18)) | Germany | To resolve the pseudo-paradox that the clinical outcome of women affected by breast cancer has improved during the last 20 years irrespective of whether they were treated in accordance with CGs or not | 1991 - 2009 | Treatment | 9 061 | Patients with primary breast cancer (BRENDA cohort) | Overall survival  Recurrence-free survival | Low |
