# Supplementary material for: Adherence to breast cancer guidelines is associated with better survival outcomes: a systematic review and meta-analysis of observational studies in EU countries
Source: BMC Health Serv Res. 2020 Oct 7;20:920. doi: 10.1186/s12913-020-05753-x (PMC7542898; doi:10.1186/s12913-020-05753-x)
Supplement: Supplementary file 5 — Additional file 5. Adherence definitions and main findings [file 12913_2020_5753_MOESM5_ESM.docx]

**Additional file 5. Adherence definitions and main findings**

| **Author(s)/ publication year** | **Adherence definition** | **Overall survival HR (95% CI)** | **Disease-free survival**  **HR (95% CI)** | **Co-variables HR** | **Costs** |
| --- | --- | --- | --- | --- | --- |
| Andreano, Rebora et al. 2017([13](#_ENREF_13)) | They defined as adherent the care pathway of patients with a proportion of met indicators ≥ 80%. | <50y: 0.88 (0.55-1.38)  50-69y: 0.68 (0.49-0.95)  >69y: 0.61 (0.48-0.77)  Cut-offs:  60%: 0.71 (0.58-0.87)  70%: 0.63 (0.52-0.76)  80%: 0.66 (0.55–0.77)  90%: 0.88 (0.73-1.07) | NR | Age, grading, stage, molecular subtype, number of nodes, Charlson index, deprivation index, marital status, deprivation, employment | NR |
| de Roos et al.([29](#_ENREF_29)) | Compliance treatment was classified as appropriate if the interventions undertaken agreed with the GLs in operation at the time of treatment. | NR | Without GL vs. with GL:4.339 (1.695 - 11.108)  GL (-) vs. GL (+): 2.778 (0.982 - 6.781) | Age, Premenopausal, Screen-detected, Family history, FNAC, SCNB, Micro calcifications, Density, Mammographic size, BCS+XRT, Axillary staging surgery, Positive margins (<1 mm), Pathological size, Grading, the time period | NR |
| Ebner, Hancke et al 2015 ([14](#_ENREF_14)) | Adherence to the German national consensus GL was defined as GL+. Treatment forms were classified as GL- if GL+ was not undertaken. | 50-69 y, any GL (-): 1.852 (1.518-2.259)  50-69 y. GL (-) RT: 3.354 (2.517-4.470)  50-69 y. GL (-) surgical: 0.911 (0.695-1.194)  50-69 y. GL (-): ET: 2.794 (2.095-3.727)  50-69 y. CT: 1.462 (1.119-1.910)  70+ y. any GL (-): 1.693 (1.392-2.060)  70+ y. GL (-) RT: 2.346 (1.881-2.926)  70+ y. GL (-) surgical: 0.599 (0.449-0.800)  70+ y. GL (-) ET : 2.260 (1.723-2.966)  70+ y. GL (-) CT: 1.371 (1.119-1.681) | 50-69 y. any GL (-): 1.752 (1.484-2.069)  50-69 y GL (-) RT: 4.280 (3.313-5.529)  50-69 y. GL (-)surgical: 0.891 (0.712-1.113)  50-69 y.ET: 3.029 (2.369-3.871)  50-69 y. GL (-) CT: 1.419 (1.129-1.783)  70+ y. any GL (-): 1.702 (1.402-2.066)  70+ y. GL (-) RT: 2.440 (1.950-3.053)  70+ y. GL (-) surgical: 0.763 (0.581-1.001)  70+ y. GL (-) ET: 2.650 (2.075-3.384)  70+ y. GL (-) CT: 1.300 (1.075-1.572) | Nodal status, tumour stage, grading, hormone receptor  status, HER2 expression, and comorbidities | NR |
| Ebner, van Ewijk et al2015([15](#_ENREF_15)) | Adherence to the German national consensus GL was defined as GL(+). Treatment forms were classified as GL(-) if GL+ was not undertaken | HR+/HER2-. any GL (-): 1.822 (1.464-2.269)  HR+/HER2-. GL (-) RT: 2.217 (1.753-2.805)  HR+/HER2-. GL (-) surgical: 0.874 (0.642-1.189)  HR+/HER2-. GL (-) ET: 2.323 (1.454-3.710)  HR+/HER2-. GL (-) CT: 2.105 (1.630-2.720)  HR+/HER2+. Any GL (-) :1.715 (0.741-3.970)  HR+/HER2+. GL (-) RT: 1.561 (0.685-3.558)  HR+/HER2+. GL (-) surgical: 0.560 (0.167-1.886)  HR+/HER2+. GL (-) ET: 1.171 (0.482-2.843)  HR+/HER2+. GL ( -) CT: 3.410 (1.063-10.939)  HR-/HER2+. any GL (-) 0.974 (0.432-2.198)  HR-/HER2+. GL (-) RT: 2.753 (1.103-6.875)  HR-/HER2+. GL (-) surgical: 0.426 (0.126-1.444)  HR-/HER2+. GL (-) CT: 1.759 (0.782-3.954)  TNBC. any GL (-): 3.694 (1.891-7.219)  TNBC. GL (-) RT: 2.748 (1.551-4.869)  TNBC. GL (-) surgical 0.401 (0.155-1.040)  TNBC. GL (-) CT: 3.801 (2.158-6.695) | HR+/HER2-. any GL (-): 1.913 (1.532-2.389)  HR+/HER2-. GL (-) RT: 2.209 (1.739-2.807)  HR+/HER2-. GL (-) surgery: 0.956 (0.702-1.301)  HR+/HER2-. GL (-) ET: 2.421 (1.872-3.131)  HR+/HER2-. GL ( -) CT: 2.083 (1.300-3.338)  HR+/HER2+. Any GL (-): 2.043 (0.868-4.809)  HR+/HER2+. GL (-) RT: 2.042 (0.910-4.586)  HR+/HER2+. GL (-) surgery: 0.499 (0.149-1.671)  HR+/HER2+. GL (-) ET: 1.721 (0.664-4.456)  HR+/HER2+. GL (-) CT: 2.468 (0.737-8.269)  HR-/HER2+. any GL (-): 0.870 (0.377-2.008)  HR-/HER2+. GL (-) RT: 2.663 (1.032-6.874)  HR-/HER2+. GL (-) surgery: 0.340 (0.099-1.172)  HR-/HER2+. GL (-) CT: 1.524 (0.668-3.476)  TNBC. any GL (-): 4.466 (2.268-8.794)  TNBC. GL (-) RT: 2.880 (1.648-5.034)  TNBC. GL (-) surgery: 0.468 (0.181-1.210)  TNBC. GL (-) CT: 4.176 (2.370-7.356) | Tumour size, nodal status, year of diagnosis, grading treatment centre, HR, HER2 and comorbidities | NR |
| Hancke, Denkinger et al. 2010([30](#_ENREF_30)) | Adherence was defined as the fulfilment of the 2005 St Gallen consensus and on the national consensus GL Breast Cancer 2004 for decisions about locoregional treatment (surgery and radiotherapy) for chemotherapy and endocrine therapy. | MA vs BCS OT ≤ 69 y: 0.99 (0.52-1.89)  MA vs BCS OT ≥ 70 y: 0.78 (0.44-1.37  MA vs BCS UT ≤69 y: 1.48 (0.89-2.45)  MA vs BCS UT ≥ 70 y: 0.84 (0.50-1.41)  RT. OT ≤69 y:: 0.16 (0.03-0.82)  RT. OT ≥70 y: 1.07 (0.33-3.47)  RT. UT ≤69 y: 3.29 (2.25-4.82)  RT. UT ≥70 y: 1.89 (1.32-2.71)  ET. OT ≤69 y: 0.66 (0.24-1.82)  ET. OT ≥70 y: 1.03 (0.22-4.71)  ET. UT ≤69 y: 1.88 (1.26-2.81)  ET. UT ≥70 y: 1.23 (0.77-1.97)  CT. UT ≤69 y: 1.52 (0.99-2.33)  CT. UT ≥70 y: 1.19 (0.78-1.82) | MA vs BCS OT ≤69 y: 0.81 (0.45-1.45)  MA vs BCS OT ≥70 y: 0.97 (0.57-1.67)  MA vs BCS UT ≤ 69 y: 1.24 (0.81-1.91)  MA vs BCS UT ≥70y: 0.99 (0.63-1.57)  RT. OT ≤69 y: 0.22 (0.06-0.80)  RT. OT ≥70 y 0.83 (0.25-2.70)  RT. UT≤69 y 3.45 (2.43-4.91)  RT. UT≥70 y 2.14 (1.53-3.01)  ET. OT ≤69 y: 0.98 (0.47-2.03)  ET. OT ≥70 y: 1.15 (0.26-5.15)  ET. UT <=69 y: 2.05 (1.45-2.90)  ET. UT ≥70 y: 1.10 (0.70-1.72)  CT. UT ≤69 y: 1.38 (0.96-2.00)  CT. UT ≥70 y: 0.85 (0.58-1.25) | Tumour stage, nodal invasion, adherence and non-adherence to adjuvant treatment, state of risk (classification of St Gallen; and  Hormone receptor. | NR |
| Jacke, Albert et al2015 ([17](#_ENREF_17)) | Quality indicators (QI) operationalised GL recommendations.  Summarising overall adherence index. Only one disrespected quality indicator devalued all possible GL-adherent indicators beforehand. | GL (-) vs. GL (+)  TI 1996-97. institutional:1.612 (0.690-3.766)  TI 1996-97 regional: 1.293 (0.500-3.348)  TI 2003-04. institutional:1.147 (0.581-2.266)  TI 2003-04. regional: 1.914 (0.772-4.745)  1996-97 vs. 2003-04  GL (+) institutional: 1.036 (0.333-3.229)  GL (+) regional: 1.922 (0.453-8.161)  GL (-). Institutional: 1.665 (1.113-2.490)  GL (-). regional: 1.196 (0.734-1.947) | NR | NR | NR |
| Mille, Roy et al. 2000 ([31](#_ENREF_31)) | Follow-up was determined to be CG-compliant or CG-noncompliant by analysing the clinical relevance of each investigation performed. | NR | NR | NR | Noncompliant follow-up cost the Social Security from 2.2 to 3.6 times more than compliant follow-up. |
| Poncet, Colin et al. 2009 ([32](#_ENREF_32)) | The adherence to the trastuzumab treatment plan French post-licensing GLs (2001), and the regional CGs (oncology care network “Convergence” Rhone-Alpes area) | Median overall survival 18.6 months  Mortality 85 /131 patients (64.9%)  70.3% 1-year survival  38.9% 2 years survival | Median progression-free survival: 7.7 months. Complete response in 5patients.  The best objective response rate after 8 cycles in both groups | NA | Cost per patient-year for treatment with trastuzumab  GL concordant 54,975€ (range 2605 - 162,093€) treatment  GL discordant treatment. 44,186€ (range 5040–224,761€) i |
| Sacerdote, Bordon et al 2013 ([33](#_ENREF_33)) | The start of the post-PGL period was 1.5 years after the introduction of PGL, as at this time they were likely to have been implemented in Piedmont hospitals. | 0.94 (0.56–1.56) | NR | Age, clinical stage and surgical unit annual case load. | NR |
| Schwentner, Wolters et al. 2012 (b) ([36](#_ENREF_36)) | German national consensus GL S3-GL. guideline adherence on the omission of any suggested adjuvant treatment or abandonment of any adjuvant treatment was classified as non-attending the suggested adjuvant therapy. | survival parameters in TNBC GL (-)  RT: 2.86 (2.24-3.64)  CT: 2.78 (2.22-3.49)  Lymph node dissection: 1.82 (1.32-2.50) | survival parameters in TNBC GL (-)  RT: 2.92 (2.24-3.81)  CT: 2.16 (1.70-2.74)  Initial surgical intervention: 2.03 (1.36-3.04) | Age, affected lymph nodes, grading, tumour size | NR |
| Schwentner, Wolters et al. 2012(a) ([35](#_ENREF_35)) | German national consensus GL S3-GL.  Omission or abandonment of any adjuvant treatment was classified as non-attending the suggested adjuvant therapy. A primary tumour (GLP) a contralateral tumour (GLC). | * Reference: 100% GL (+)  1-2 GV-: 2.27 (0.97-5.35)  ≥3 GV: 3.71 (1.62-8.46)  *Reference: GLP (+) - GLC (+)  GLP (-)-GLC (+): 1.57 (0.29-8.59)  GLP (+) -GLC (-): 2.83 (0.55-14.61)  GLP (-)-GLC (-): 6.98 (1.69-28.73) | * Reference: 100% GL (+)  1-2 GV: 2.34 (1.10-4.98)  ≥3 GV: 2.66 (1.25-5.67)  *Reference: GLP (+) – GLC (+)  GLP (-)-GLC (+): 3.06 (0.65-14.47)  GLP (+)-GLC (-): 4.20 (0.87-20.24)  GLP (-)-GLC (-): 7.45 (1.81-30.69) | Tumour size, nodal status, grading and age | NR |
| Schwentner, Wockel et al 2013([34](#_ENREF_34)) | German national consensus GL (S3 GL) for the decision of loco-regional treatment (surgery and radiotherapy), chemotherapy, and endocrine therapy (only non-TNBC) | TNBC  ≥65 y: 2.89 (p=0.001))  50–64 y: 1.27 (p=0.515)  <50 y: 3.47 (p=0.001)  * ≥1 GLV vs. 100% GL (+)  GL (+) was significantly lower in the  TNBC patients compared to the non-TNBC population (P < 0.001) | TNBC  ≥65 y: 2.72 (p=0.001)  50–64 y: 1. 16 (p = 0.633).  <50 y: 3.20 (p < 0.001);  * ≥1 GLV vs. 100% GL (+) | Tumour size, grading, nodal status (positive nodes), menopausal status , year of diagnosis, comorbidities and age | NR |
| van de Water, Bastiaannet et al. 2012([37](#_ENREF_37)) | Non-adherence was defined as undertreatment (omission of recommended treatment) or overtreatment (administration of treatment that was not recommended). | GL (+) (5-year survival)  <100% <65 y: 1.75 (1.50-2.05)  <100% ≥75y: 1.62 (1.41-1.85)  Reference: 100% GL(+) | NR | Histological grade, tumour category, node status estrogen receptor status  progesterone receptor status and age |  |
| Van Ewijk, Wockel et al 2015 ([38](#_ENREF_38)) | German national consensus GL [S3-GL, 2008 Omission or abandonment of any indicated adjuvant treatment was classified as non-compliance. | *Reference: <65 y  GL (+) 65-80 y: 1.34 (1.00-1.81)  GL (-) 65-80 y: 2.50 (1.87–3.33) | *Reference: <65 y  GL (+)65-80 y: 1.17 (0.91-1.50)  GL (-) 65-80 y 2.10 (1.61–2.62) | Year of diagnosis, a patient treated at university department, nodal status, grading, tumor size, hormone receptor status, menopausal status, erb-2-status, and comorbidities, | NR |
| Wimmer Theresa et al.  2019([43](#_ENREF_43)) | Adherence to German S3 guidelines for breast cancer and their quality indicators. Adherence was defined as the use of radiotherapy after breast-conserving therapy in invasive breast cancer. | 0.64 (0.46-0.88) | Local recurrence-free survival  0.20 (0.16-0.26) | Age at diagnosis, grading, tumour size, nodal status, stage of tumour, HER2 status, hormone receptor status, lymphatic/venous invasion, endocrine therapy, immunotherapy, and chemotherapy | NR |
| Varga, Wischnewsky et al. 2010 ([39](#_ENREF_39)) | GL adherence was defined in the German Cancer Society in 2004. Treatment conforming: no deviation from the recommendations. | 4.73 (1.08-20.69) | 2.95 (1.11-7.83) | Tumour stage, nodal status, histology grade, the risk of recurrence, lymphovascular invasion, endocrine responsiveness, year of diagnosis and adjuvant treatment | NR |
| Wockel, Kurzeder et al. 2010 ([41](#_ENREF_41)) | Consensus S3 GL German Cancer Society in 2004.  Separate forms of GL-adherent cytostatic therapies: St. Gallen covenants and risk classifications 2004.  Non-adherent therapies: over- and under therapies. | Reference: 100% GL(+)  1-2 GV: 2.44 (1.85-3.21)  >=3 GV: 4.34 (2.90-6.50)  Effect of GL(+)  RT: 3.61 (2.75-4.75)  CT: 2.59 (2.02-3.31)  ET: 1.75 (1.28-2.40)  BCT: NR  Axillary dissection: 1.47 (1.02-2.12) | Reference: 100% GL(+)  1-2 GV: 2.04 (1.60-2.60)  >=3 GV: 3.64 (2.43-5.45)  Effect of GL(+)  RT : 3.00 (2.30-3.92)  CT: 2.12 (1.67-2.69)  ET: NR  BCT: 1.88 (1.25-2.83)  Axillary dissection NR | Tumour size, nodal status, grading |  |
| Wockel, Varga et al.2010 ([40](#_ENREF_40)) | Consensus S3 GL German Cancer Society in 2004. Separate forms of GL-adherent cytostatic therapies: St. Gallen covenants and risk classifications 2004.  Non-adherent therapies: over and undertherapies. | Effect of GL (+)  RT : 3.68 (2.92-4.64)  CT: 2.15 (1.74-2.65)  ET: 1.30 (1.01-1.68)  BCT: 1.49 (1.07-2.07) | GL (+) vs. GL (-) therapy  TI 1992-2000 2.51 (1.88-3.35)  TI 2001-2005: 2.05 (1.46-2.92)  Effect of GL (+)  RT: 2.39 (1.84-3.12)  CT: 2.22 (1.79-2.75)  ET: 1.72 (1.33-2.21)  BCT: 1.64 (1.18-2.27) | Age, tumour size, nodal status and grading | NR |
| Wockel, Wolters et al. 2014 ([42](#_ENREF_42)) | German national consensus GL (S3 GL). Omission of or the abandonment of any adjuvant treatment was classified as noncompliance | Adjuvant RT adjusted: 0.23 (0.20-0.26)  Effect of GL (+)  CT: 0.671 (0.588-0.765)  RT: 0.638 (0.551-0.740)  ET: 0.617 (0.527-0.723)  BCT: 0.647 (0.565-0.739) | Adjuvant RT adjusted: 0.23 (0.15-0.34)  Effect of GL (+)  CT: 0.519 (0.447-0.603)  RT: 0.574 (0.482-0.682)  ET: 0.605 (0.506-0.723)  BCT: 0.789 (0.669-0.930) | Age, affected lymph nodes, grade, hormone  receptor status, menopause status, year of diagnosis, treatment  in a university hospital, tumour size, erbB-2-status, and comorbidities. |  |
| Wollschlager, Meng et al. 2017([16](#_ENREF_16)) | German national consensus GL Omission of any suggested adjuvant treatment was classified as noncompliance. | 1.65 (1.33-2.07) | 1.84 (1.53-2.22) | Age, comorbidity and tumour characteristics | NR |
| Wolters, Wischhusen et al. 2015 ([18](#_ENREF_18)) | German national consensus GL (S3 GL) 2008 for the adjuvant treatment of breast cancer | TI (1991-2000): 0.47 (0.36-0.62)  TI (2001-2009): 0.48 (0.41-0.57)  overall: 0.46 (0.40-0.53)  *100% GL (+)therapy vs. not treated in accordance with GLs | TI (1991-2000): 0.49 (0.37-0.66)  TI (2001-2009): 0.58 (0.49-0.68)  Overall: 0.51 (0.44-0.59)  *100% GL (+) therapy vs. not treated in accordance with GLs | Tumour grade, hormone receptor status, affected lymph nodes, menopause status, year of diagnosis, age, and comorbidities | NR |

NR: non reported, NA: not applicable; BCT: breast-conserving therapy; CT: chemotherapy; ET: endocrine therapy (hormonal therapy); GL: guideline; GL (+): guideline adherent GL (-): guideline non-adherent; GV: guideline violation; GLP: primary tumour, GLC: contralateral tumour;HR: hormone receptor;. MA: mastectomy; OT: overtreatment; RT: radiotherapy, TI: time interval; UT: undertreatment; y: years, TNBC: triple negative breast cancer
